# Discovery of Pyrrolidine-2,3-diones as Novel Inhibitors of *P. aeruginosa* PBP3

**DOI:** 10.3390/antibiotics10050529

**Published:** 2021-05-04

**Authors:** Arancha López-Pérez, Stefan Freischem, Immanuel Grimm, Oliver Weiergräber, Andrew J. Dingley, María Pascual López-Alberca, Herbert Waldmann, Waldemar Vollmer, Kamal Kumar, Cuong Vuong

**Affiliations:** 1 AiCuris Anti-Infectives Cures GmbH, 42117 Wuppertal, Germany; arancha.lopez@aicuris.com (A.L.-P.); immanuel.grimm@Aicuris.com (I.G.); kamal.kumar@aicuris.com (K.K.); 2 Centre for Bacterial Cell Biology, Biosciences Institute, Newcastle University, Newcastle upon Type NE2 4AX, UK; waldemar.vollmer@newcastle.ac.uk; 3 Institute of Biological Information Processing, Structural Biochemistry, Forschungszentrum Jülich, 52428 Jülich, Germany; s.freischem@fz-juelich.de (S.F.); o.h.weiergraeber@fz-juelich.de (O.W.); a.dingley@fz-juelich.de (A.J.D.); 4 Institut für Physikalische Biologie, Heinrich-Heine-Universität Düsseldorf, 40225 Düsseldorf, Germany; 5 Max Plank Institute of Molecular Physiology, Department of Chemical Biology, 44227 Dortmund, Germany; mpascualla@gmail.com (M.P.L.-A.); herbert.waldmann@mpi-dortmund.mpg.de (H.W.)

**Keywords:** PBPs, *P. aeruginosa*, penicillin binding proteins, HTS, β-lactams, multi-drug resistance, antibiotics, antibacterial agents, drug discovery and screening

## Abstract

The alarming threat of the spread of multidrug resistant bacteria currently leaves clinicians with very limited options to combat infections, especially those from Gram-negative bacteria. Hence, innovative strategies to deliver the next generation of antibacterials are urgently needed. Penicillin binding proteins (PBPs) are proven targets inhibited by β-lactam antibiotics. To discover novel, non-β-lactam inhibitors against PBP3 of *Pseudomonas aeruginosa*, we optimised a fluorescence assay based on a well-known thioester artificial substrate and performed a target screening using a focused protease-targeted library of 2455 compounds, which led to the identification of pyrrolidine-2,3-dione as a potential scaffold to inhibit the PBP3 target. Further chemical optimisation using a one-pot three-component reaction protocol delivered compounds with excellent target inhibition, initial antibacterial activities against *P. aeruginosa* and no apparent cytotoxicity. Our investigation revealed the key structural features; for instance, 3-hydroxyl group (R^2^) and a heteroaryl group (R^1^) appended to the *N*-pyrroldine-2,3-dione via methylene linker required for target inhibition. Overall, the discovery of the pyrrolidine-2,3-dione class of inhibitors of PBP3 brings opportunities to target multidrug-resistant bacterial strains and calls for further optimisation to improve antibacterial activity against *P. aeruginosa*.

## 1. Introduction

*Pseudomonas aeruginosa* is a ubiquitous Gram-negative bacterium and an opportunistic pathogen that causes both acute and chronic infections in humans [1,2]. *P. aeruginosa* is a major cause of nosocomial infections in immunocompromised and critically ill patients treated in intensive care units (ICUs) and the main cause of infections with a high mortality rate in cystic fibrosis patients. Furthermore, *P. aeruginosa* is a leading cause of infections that arise from wounds and burn injuries, and community-acquired and ventilator-associated pneumonias [3]. Notably, *P. aeruginosa* is intrinsically resistant to most antibiotics because of several multidrug efflux pumps, the low permeability of the cell envelope and its ability to adapt to unfavourable growth or environmental conditions (especially during infections) by virtue of its large and versatile genome [4]. The World Health Organisation (WHO) has recently categorised *P. aeruginosa* as a leading and critical bacterium in its priority pathogen list, in order to accelerate the development of innovative antibacterials against this pathogen [5]. Thus, the discovery of new small molecules and potentially novel mechanism of action on a target represents a key strategy to combat *P. aeruginosa* infections and break the current resistance mechanisms against available antibiotics.

Bacterial cell shape is maintained through the essential peptidoglycan (PG) layer of the cell wall. The PG plays a key role in the growth cycle and protects the bacterial cell from bursting by turgor, which prevents cell lysis and, eventually, death [6,7,8]. Bacteria possess sophisticated mechanisms to maintain the integrity and biogenesis of the PG layer as well as coordinate the synthesis of the PG layer during the life cycle of the cell [6,7,8]. PG is a net-like polymer consisting of glycan strands made of alternating, β-1-4-linked *N*-acetylglucosamine and *N*-acetylmuramic acid residues, which are linked by short peptides. Glycosyltransferases (GTases) polymerise the glycan strands from lipid II precursor. DD-transpeptidases (TPases) belonging to the family of penicillin binding proteins (PBPs) form the peptide cross-links [6,7,8]. Class A PBPs are bifunctional GTase/TPases, while the class B PBPs are monofunctional PG transpeptidases [6,7,8,9,10,11]. Class C PBPs do not form DD-crosslinks but display DD-endopeptidase or DD-carboxypeptidase activity. Additional monofunctional GTases that associate with the monofunctional class B PBPs have been described as member of the SEDS family (e.g., FtsW and RodA) [9,12]. The GTase and TPase activities are essential for bacterial survival, and the inhibition of these activities leads to cell death.

The TPase catalytic signature contains nine conserved residues in three motifs: S*XXK in the *N*-terminal part of helix α2, SXN in the α4-α5 linker and KS/TG in the strand β3 [11,13]. The nucleophilic serine (S*) attacks the carbonyl carbon of the D-alanine at position 4 of the donor pentapeptide substrate to transfer the tetrapeptide to the ε-amino group of a *meso*-diaminopimelic acid (*m*DAP) residue at position 3 of an acceptor peptide. The TPase domain of each PBP is highly conserved, but different bacteria have varying numbers of PBPs, and these PBPs can have different roles. Gram-negative species, such as *Escherichia coli* or *P. aeruginosa* (which have been studied extensively), harbour more than eight PBPs, of which some are essential and validated antibacterial targets for β-lactam antibiotics. Gram-negative rod-shaped bacteria require at least one class A bifunctional TPase/GTase, PBP1A or PBP1B and the monofunctional TPases PBP2 and PBP3 to growth and survive [11,13].

PBPs are targeted by β-lactam antibiotics. However, the efficacy of β-lactams has dramatically decreased with the emergence and spread of multidrug-resistant (MDR) strains harbouring a vast range of genetically or plasmid-encoded β-lactamases and additional resistance mechanisms (e.g., efflux transporters and target mutations). β-lactamases abolish the antibacterial activity of β-lactam antibiotics by hydrolysing the β-lactam ring. A further mechanism to bypass the effect of β-lactams is the acquisition of alternative PBPs encoded in horizontal-genetic elements or by mutations in the chromosomal genes encoding PBPs (e.g., the *mecA* gene encoding PBP2a in *Staphylococcus aureus*) [14,15]. Interestingly, β-lactams are no longer the only chemical class capable of inhibiting PBPs. The discovery of cyclic boronates and diazabicyclooctanes (DBOS) demonstrate that the TPase domain can be inhibited by mechanisms distinct from the known binding mode of β-lactam [16,17,18]. Therefore, the discovery of other non-β-lactam PBP-targeting antibiotics may lead to the development of new, urgently needed antibacterials that will not be inactivated by β-lactamases.

In this study, we selected PBP3 of *P. aeruginosa* (*^Pa^*PBP3) for a target-based inhibitor screening to discover novel chemical scaffolds. Because *^Pa^*PBP3 is essential for the survival of *P. aeruginosa*, this protein represents a key target for antibacterial drug discovery and development. It is a clinical validated target, and the absence of a human counterpart reduces the risk of drug-related side effects. The catalytic domain of PBP3 is located in the periplasm, making it accessible to potential small molecule inhibitors. Furthermore, we report the optimisation of the previously developed S2d assay to measure the TPase activity of *^Pa^*PBP3. Using the optimised assay, we screened a library of 2455 compounds and identified novel potent PBP3 inhibitors derived from the pyrrolidine-2,3-dione scaffold that target Gram-negative bacterial, especially *P. aeruginosa*.

## 2. Results

### 2.1. Optimisation of the S2d Assay for Fluorescence-Based Screening

**Figure 1:**
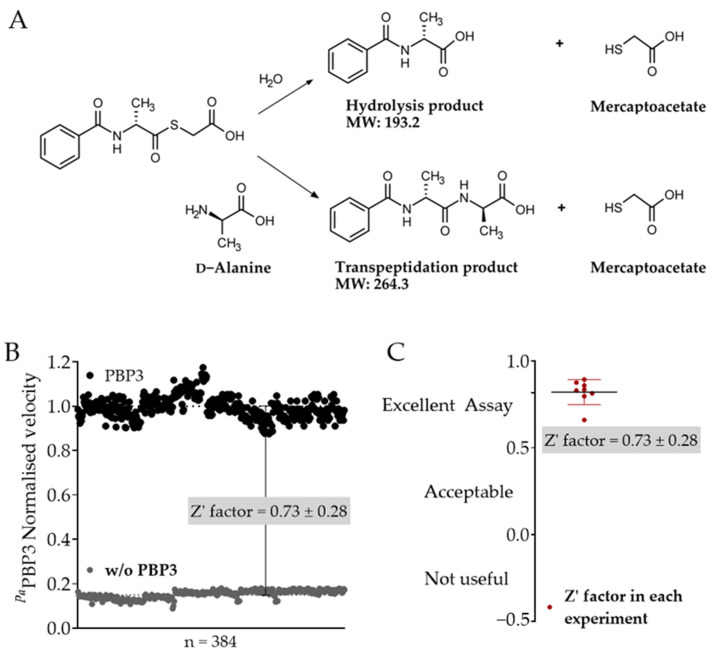
Transpeptidation biochemical assay. (**A**) Enzymatic reaction. The thiol substrate is used as an acyl donor for the transpeptidation reaction in the presence of carbonyl acceptors (i.e., d-alanine). The reaction yields a transpeptidation product and a mercaptoacetate. The thiol group in the mercaptoacetate enables the indirect measurement of the TPase reaction by its quantification with a thiol dye. Figure adapted from [25]. (**B**) Fluorometric S2d-based assay for *^Pa^*PBP3. The Z’factor was determined using the average and SD of the positive (black dots: +PBP3) and negative (grey dots: without (w/o) PBP3). (**C**) The Z’-factor of the fluorometric S2d-based assay for *^Pa^*PBP3. Red dots represent the Z’factor calculated for each plate measured and the Z’factor indicated and highlighted in grey was calculated using the data from eight replicates.

Thioesters of hippuric acids have been previously used in the presence of a carbonyl acceptor (e.g., d-alanine) as an artificial substrate for measuring the TPase activity of several PBPs, as illustrated in Figure 1A. The TPase reaction can be measured by quantifying the transpeptidation product using LC-MS/MS or by quantifying the released mercaptoacetate product using thiol dyes [19,20,21,22,23].

Traditional colorimetric dyes such us aldrithiol (2,2’dipyridyldisulfide) or Ellman’s reagent (5,5’-dithiobis-(2-nitrobenzoic acid)) have been used to investigate PBPs kinetics (e.g., PBP1B [24], PBP3 [22] and PBP5 [25] of *E. coli*, PBP2B [22] and PBP2x [19,25,26,27] of *Streptococcus pneumoniae*, PBP1 and PBP3 of *Enterococcus hirae* [22], the DD-peptidase R61 of *Streptomyces* [20] and the DD-peptidase R39 of *Actinomadura* sp. R39 [18,28]) and for quantifying the effect of protein partners on the activity of *E. coli* PBP1B [29]. However, colorimetric readouts limit the use of the assay in drug discovery because the intrinsic spectra of many test compounds overlap with that of aldrithiol or Ellman’s reagent. Therefore, we replaced the colorimetric dye with a fluorescent thiol detecting dye, monobromobimane (mBBr) (Ex/Em: 394/490 nm). The emission spectrum of mBBr does not interfere with the absorbance wavelength of most common compounds, thus improving the quality of the read-out spectra.

Initial experiments were performed to determine the optimal conditions, which were found to be 0.3 µM *^Pa^*PBP3, 0.8 mM artificial substrate S2d, 1 mM D-alanine and 0.05 mM thiol dye. The assays were carried out in the presence of 5% DMSO and 0.01% Triton X100 to avoid the formation of colloidal structures and adsorption of protein and/or compounds to surfaces [30,31]. The Z’-factor determined for the assay was 0.73 ± 0.28, indicating the suitability and robustness of the assay for high throughput screening (HTS) [29,32] (Figure 1B,C). Additional statistical parameters, including the signal-to-noise ratio and the coefficient of variation and their values, indicated that the assay was sensitive and robust (Table S1).

The newly modified fluorescence-based S2d_fluo_ assay was then employed in a HTS campaign using a focused library consisting of 2455 pre-selected cysteine protease-targeting inhibitors (ChemDiv, San Diego, USA) to identify potential *^Pa^*PBP3 inhibitors. Screening of the library identified 27 hits that inhibited PBP3 over a range of 60% to 100% at a concentration of 100 µM (Table 1 and Table S2). Interestingly, all potent PBP3 inhibitors contained a pyrrolidine-2,3-dione core, indicating a potential conserved scaffold that could be used as a new starting point for the further development of PBP3 TPase inhibitors. Three proprietary Max Plank Institute of Molecular Physiology (MPI) compounds containing the pyrrolidine-2,3-dione-like core were also added to the hit selection (Table 1).

To further characterise the inhibitory activity of the 30 hits, we performed concentration-dependent experiments using the S2d_fluo_ assay and compounds between 100 µM and 1.56 µM. Table 1 shows nine compounds that inhibited the PBP3 enzyme in a concentration-dependent manner. Notably, compound **2** exhibited an excellent potency, with an IC_50_ of 4 ± 6 µM in the S2d_fluo_ assay. The remaining 21 compounds did not show concentration-dependent inhibition but had only an effect at the highest test concentrations of 100 µM (Table S2), suggesting nonspecific inhibition. The IC_50_ values of the test compounds in the S2d_fluo_ assay varied from 4 µM to >100 µM and only compounds **1**, **2**, **3** and 4 inhibited the enzyme with IC_50_ < 50 µM. This concentration was the second highest concentration used in the assay and was the selected threshold for hits selection.

Bocillin FL is a fluorescent penicillin V derivate used as a competitor for PBP inhibitors [32,34,35,36]. To further validate the hits, we measured the fluorescence polarisation (FP) of Bocillin FL in the presence of decreasing concentrations of compounds **1** to **9** (Table 1 and Table S2). Because of the low potency of these compounds when compared with that of other covalent PBP inhibitors (such as β-lactams with IC_50_ values in the nM concentration range), we incubated the inhibitors for 30 min prior to the addition of Bocillin FL. Seven of the nine compounds competed for the TPase site and inhibited the binding of Bocillin FL in a dose-dependent manner, indicating a potential occupation of the active site. In contrast, compounds **3** and **4** showed IC_50_ values of 19 ± 1 µM and 24 ± 20 µM in the S2d_fluo_ assay but failed to inhibit Bocillin FL binding at test concentrations. These data suggested that compounds **3** and **4** were presumably bound to *^Pa^*PBP3 at a site distinct from the TPase pocket in a way that still affects the enzyme activity by an unknown mechanism.

Based on the observed target inhibition and supported by the in vitro biochemical data, we selected compounds **1** and **2** as promising starting points for the design and synthesis of a pyrrolidine-2,3-dione focused library of small analogues. Compound **1** was used as the primary scaffold for optimisation because of the simplicity of its structure and the relative weak activity in other molecules with *N*-aryl pyrrolidine-2,3-diones (2–5, Table 1). The design of this focused library aimed to: (i) preliminarily explore the structure–activity relationship (SAR) of the compounds and (ii) confirm the inhibitory target activity of this compound cluster. Notably, small molecules with a pyrrolidine-2,3-dione core have been identified previously in a virtual screen as potential inhibitors of tyrosine phosphatase B (MptpB) from *Mycobacterium tuberculosis.* However, the inhibition activities reported for small molecules that are similar to the hits identified here were lower than 40% and thus did not justify further studies [37]. Among other known biological activities, pyrrolidine-2,3-dione molecules are reported as stabilisers of the potential 14-3-3-PMA2 protein–protein interaction of *Nicotiana tabacum* with a *K*_d_ of 80 µM [38,39]. Thus, we discovered herein a novel biological potential of the pyrrolidine-2,3-dione class of compounds and pursued their further development as an innovative antibacterial class with a new mechanism of action against *^Pa^*PBP3.

### 2.2. Synthesis of Compound **1** Analogues and Generation of the Pyrrolidine-2,3-dione Cluster

**Figure 2:**
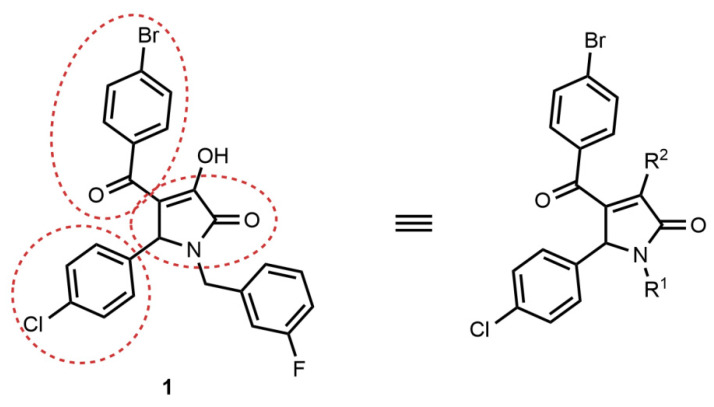
PBP3 targeting substituted pyrrolidin-2,3-diones.The pyrrolidine-2,3-dione core was modified at the nitrogen (R^1^) and at position 3 (R^2^). The rest of the molecule was maintained in the pyrrolidine-2,3-dion derivatives.

**Scheme 1:**
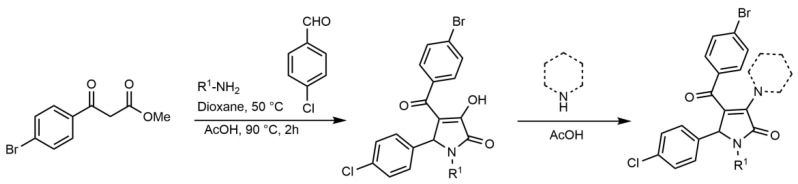
Synthesis of pyrrolidin-2,3-diones. One-pot three-component reaction used to synthesise the pyrrolidine-2,3-dione derivatives.

Inspection of the structures of the initial list of inhibitory and non-inhibitory pyrrolidine-2,3-dione compounds (Table 1) led us to conclude that the best active molecules embody a 3-hydroxy-2,5-dihydro-1H-pyrrol-2-one core with three substitutions. We observed that halogen substituted benzoyl- and phenyl groups were required for activity at positions 4 and 5, respectively, of the core scaffolds (Figure 2). Therefore, the pyrrolidine-2,3-dione core was modified at the nitrogen (R^1^) and at position 3 (R^2^), and, thus, 15 analogues were designed and synthesised (Scheme 1 and Table 2).

**Figure 3:**
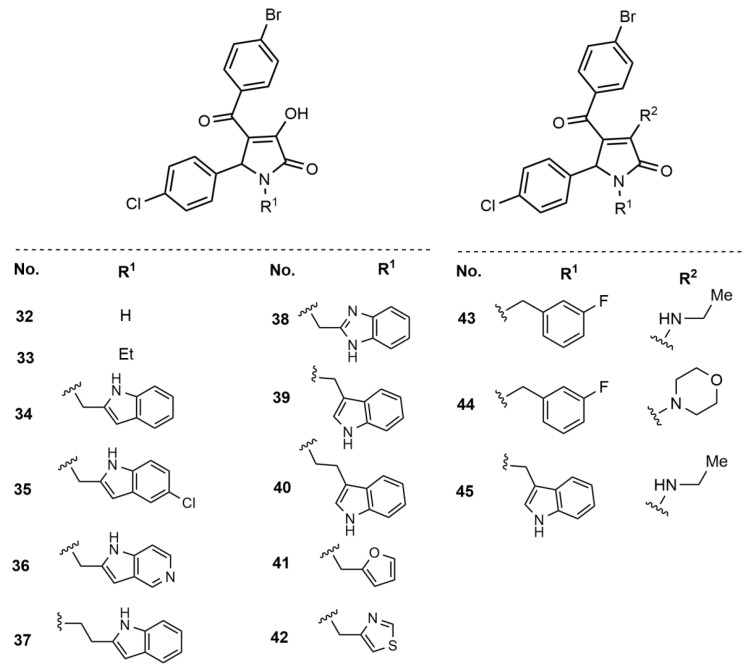
Pyrrolidine-2,3-derivatives. The pyrrolidine-2,3-dione core was modified at the nitrogen (R^1^), whereas the hydroxyl substitution at position 3 (R^2^) was maintained to generate compounds **32**–**42**. Position 3 (R^2^) was substituted in analogues **43**–**45** to investigate the essentiality of the hydroxyl group.

The desired pyrrolidine-2,3-diones were prepared using a one-pot three-component reaction protocol reported previously (Scheme 1) [40]. In a typical reaction procedure leading to pyrrolidine-2,3-diones with R^1^ substitution, a mixture of amine (R^1^NH_2_) (1.0 mmol) and aldehyde (1.0 mmol) in dry dioxane (2 mL) was stirred for 30 min at 50 °C. Then acetic acid (0.5 mL, 8.74 mmol) and p-bromophenyl pyruvate (1.0 mmol) were added and the resulting mixture was stirred for 2 h at 90 °C. After cooling the reaction mixture to ambient temperature, the precipitates were filtered, washed with dioxane and dried in vacuo to yield the target compounds with a 3-hydroxy group. Different N-substitutions (heterocycles in particular) were bound to the pyrrolidone via alkyl linkers that were also explored, considering the desired physical chemical properties of the final compounds. To this end, fluoro-substituted benzyl groups, benzylic five-membered heterocycles (furan and thiazole) and benzimidazole and indoles were linked to the core structure via methylene or ethylene linkers (Figure 3).

Treatment of the 1,4,5-substituted 3-hydroxy-pyrrolidines with ethyl amine or morpholine amine in an acidic solvent yielded the pyrrolidines with corresponding amine substitutions at position 3 (Figure 3).

Next, heterocyclic groups were introduced at the N-benzylic position as R^1^ to generate more polar compounds with better logP and hydrogen-bond donor/acceptor profiles (Figure 3). Pyrrolidones attached to the indole and aza-indole ring via C2-connection and by a methylene linker (**34**–**36**) or ethylene linker (**37**) were synthesised following the above-mentioned method (Scheme 1). The indole ring was replaced by benzimidazole and other heterocycles (**38**). More indole-rings linked via the C3-connection and through methylene (**39**) and ethylene (**40**) linkers were also obtained. Small heterocycles, such as furan (**41**) and thiazole (**42**), were introduced as R^1^ substitutions via a methylene linker. As a control to study the role of N-substitution on the biological activity of this class of small molecules, an unsubstituted pyrrolidone (**32**) and the simpler N-ethyl pyrrolidone (**33**) were synthesised. Finally, a subset of this class of pyrrolidones was synthesised wherein the 3-hydroxyl function was replaced by either ethylamine or morpholine (**43**–**45**).

### 2.3. Inhibition of ^Pa^PBP3 by Pyrrolidine-2,3-dione Derivates

Initially, we investigated the inhibitory effect of pyrrolidine-2,3-derivates on the TPase activity of *^Pa^*PBP3 (Table 2) by determining the IC_50_ using the biochemical assays with S2d_fuo_ or FP-Bocillin FL. The results are summarised in Table 2.

Comparing the inhibitory activities of the new compounds revealed two key structural properties that are required for the inhibition of the *^Pa^*PBP3: the 3-hydroxyl group substitution (R^2^) and a bulky group such as a benzyl substitution or a heteroaryl group at the R^1^ position. Replacement of the 3-fluorophenylgroup with a hydrogen atom (analogue **32**) or an ethyl group (analogue **33**) caused loss of the inhibitory effect, indicating that a benzylic group engaged with *^Pa^*PBP3, most likely through a hydrophobic interaction with the active site of the target enzyme. In line with these results, when the benzyl group was substituted with some benzylic heterocycles (for example, an indole in analogue **34**), we observed a two-fold increase in inhibition (IC_50_ = 14 µM) when compared with that of the parental compound **1** (IC_50_ = 28 µM). Substitution of the methyl indole with ethyl thiazole in analogue **42** or a furan in analogue 41 abolished the inhibitory activity. Finally, we explored whether the position wherein a heterocycle is linked to the *N*-pyrrolidinone influences the activity by synthesizing and testing analogues **38** and **39**. The additional nitrogen group in the benzimidazole resulted in a drastic loss of the inhibitory activity in by **38** and the C3-linkage of the indole moiety in **39** increases the IC_50_ values to 24 µM. Interestingly, a similar C3-linked indole (**40**) attached via an ethylene linker displayed an improved target inhibition of 8 μM, probably due to better fitting of the heterocycle into the binding site of *^Pa^*PBP3. The inhibitory potential of the corresponding C2-appended indole (**37**) was almost reduced by two when compared with that of **40**, indicating a narrow structure-activity space to fit the ligand into binding site of the protein. In fact, the IC_50_ displayed by **37** was 16 µM and very similar to that of the analogue **34** (IC_50_ = 14 µM). A pyrrolidone with 5-chloro substitution emerged as another interesting candidate with an improved IC_50_ value of 3 µM, suggesting that the chloride atom interacts with the protein. All pyrrolidones where R^2^ was not a hydroxyl group and was replaced by amines (**43**–**45**) lost their inhibitory effect on the activity of *^Pa^*PBP3 and the Bocillin FL bound to the TPase site. These results strongly suggest that the hydroxyl group plays an important role in recognising and/or interacting with *^Pa^*PBP3.

The IC_50_ values obtained by the FP assay (Table 2) correlated well with the IC_50_ values obtained by the S2d_fluor_ assay, indicating that the compounds competed for the same catalytic TPase pocket site occupied by Bocillin FL as a representative β-lactam analogue to exert target inhibition, which eventually causes bacterial cell death.

**Figure A1:**
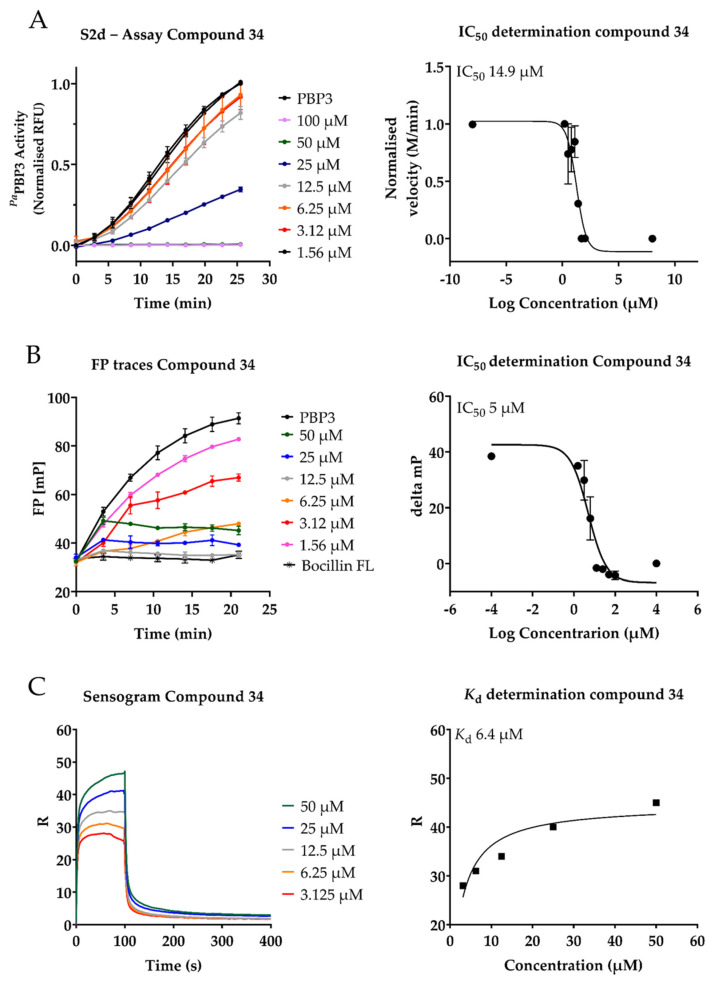
Biochemical characterisation compound **34** as *^Pa^*PBP3 inhibitor. (**A**) Fluorescence −S2d based assay, the left panel represent the *^Pa^*PBP3 kinetic curves in the presence of decreasing concentration of compounds. The right panel shows the IC_50_ normalised. (**B**) Fluorescence Polarisation assay. The left panel represents the raw bocillin FL fluorescence polarisation traces in the presence of decreasing concentration of compounds and the right panel the calculated IC_50_. (**C**) SPR assay. The left panel shows the sensorgrams signals in the presence of compound and the right panel the *K*_d_ determination.

**Figure A2:**
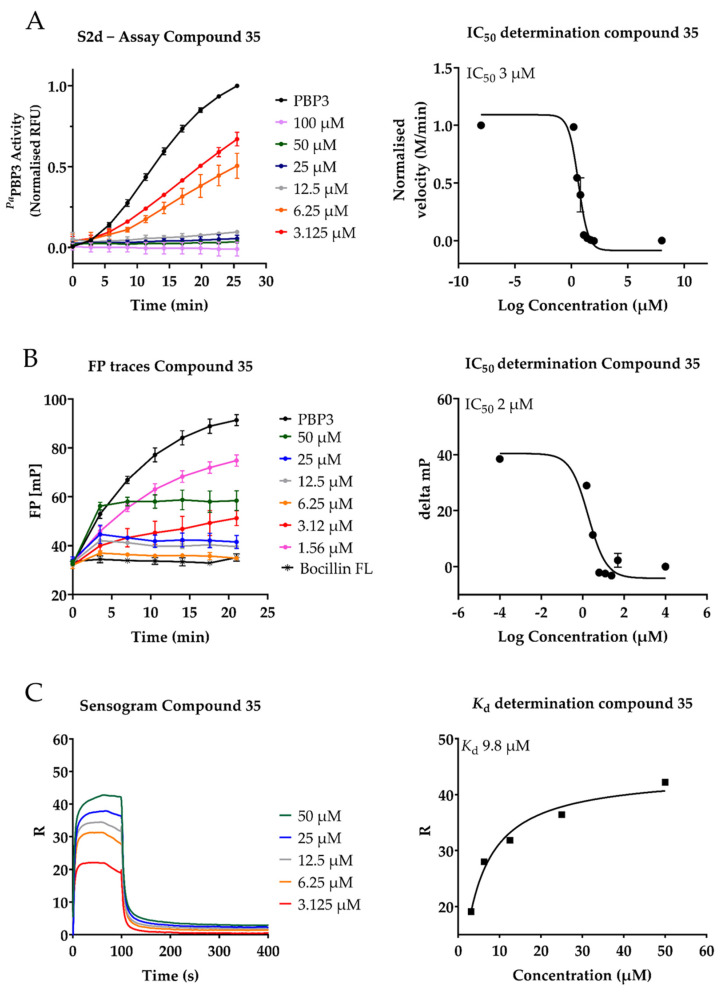
Biochemical characterisation compound **35** as *^Pa^*PBP3 inhibitor**.** (**A**) Fluorescence − S2d based assay, the left panel represent the *^Pa^*PBP3 kinetic curves in the presence of decreasing concentration of compounds. The right panel shows the IC_50_ normalised. (**B**) Fluorescence Polarisation assay. The left panel represents the raw bocillin FL fluorescence polarisation traces in the presence of decreasing concentration of compounds and the right panel the calculated IC_50_. (**C**) SPR assay. The left panel shows the sensorgrams signals in the presence of compound and the right panel the *K*_d_ determination.

Next, we confirmed the binding of the best inhibitors (analogues **34**, **35**, **40**, **38** and **37**) by surface plasmon resonance (SPR), using *^Pa^*PBP3 coated on a CM5 chip as the ligand. The sensorgrams (Figure A1C and Figure A2C) indicated fast association and dissociation with all compounds tested. Dissociation constants (*K*_d_) were determined via steady-state analysis using the maximum resonance signal obtained with a range of analyte concentrations. The calculated *K*_d_ values of the investigated compounds ranged from 6.4 to 62.7 µM (Table 2). These results indicate that, in general, the compounds bind to the protein at concentrations on the order of the IC_50_. The *K*_d_ value obtained for compound **37** was too high to conclude a specific binding, although the IC_50_ values calculated by both the S2d_fluor_ and the Bocillin FL assay indicated a good inhibition.

### 2.4. Antimicrobial Activities of Pyrrolidine-2,3-dione Derivates

The results presented above implied that seven pyrrolidine-2,3-dione derivatives inhibited the activity of *^Pa^*PBP3 in vitro. Consequently, we were very interested in investigating the potential of these compounds to affect the growth of *P. aeruginosa*. Two *P. aeruginosa* bacterial strains (the wild-type PAO1 and the isogenic ΔmexB ΔmexX ΔmexCD-oprJ efflux pump mutant) were used to determine the MIC and the potential role of the main *P. aeruginosa* efflux systems in compound uptake [34,35]. To increase the antibacterial sensitivity of the tested *P. aeruginosa* isolates to the potential inhibitors, we performed MIC experiments in the presence and absence of a sub-MIC concentration (4 µg/mL) of the outer membrane permeabilising polymyxin B nonapeptide (PMBN). The tested compounds showed only antibacterial activities in the presence of PMBN, and no growth inhibition was detected in the absence of PMBN at the highest compound concentration of 100 µM (data not shown). Thus, we concluded that the physicochemical properties of the pyrrolidine-2,3-dione compounds were perhaps not yet favourable enough to cross the *P. aeruginosa* cell envelope without a permeabiliser because of their high hydrophobicity.

The results in Table 2 indicated that analogues **34**, **35**, **37** and **39** inhibited the growth of *P. aeruginosa* wild-type PAO1 and the efflux mutant in the presence of PMBN. Interestingly, these results showed a direct correlation between target inhibition and, eventually, an antibacterial effect. Surprisingly, the MICs of compounds **34**, **35** and **39** were 2 to > 4-fold lower against the wild type PAO1 when compare with the efflux mutant K2896. This effect was even more pronounced with compound **40**, which was not active against the mutant but was active against the wild type strain. In contrast, **compounds** **1** and **41** showed MICs against the *P. aeruginosa* K2896 of 12.5 µM and 25 µM, respectively, while no inhibitory activity was detected against PAO1, suggesting that these two compounds were prone to efflux by the Mex efflux system. Notably, compound **39** did not inhibit *^Pa^*PBP3 in vitro. Thus, its antibacterial effect is likely caused by an alternative mechanism.

Additional bacterial strains were tested, and the MIC values are summarised in Table S3. Overall, the antimicrobial activity of the most active compounds correlated between the in vitro inhibition of *^Pa^*PBP3 and the measured MIC values, especially against the wild-type *P. aeruginosa* PAO1 strain.

### 2.5. Effect of the Pyrrolidine-2,3-diones on Eukaryotic Cells

We finally tested the cytotoxicity of the compounds against four eukaryotic cell lines (Table 2 and Table S5), NRK-52E, an epithelial kidney cell line of *Rattus norvegicus*; Hep G2, an human epithelial liver cell line; and two suspension cell lines: CCRF-CEM, a T lymphoblast; and H9, a cutaneous T lymphocyte cell line. Most *^Pa^*PBP3 inhibitors were found not to be cytotoxic toward the tested concentrations, except for compounds **36**, **37** and **45** that showed high cytotoxic potential at the tested cell lines, which is most relevant for compound **37**, with an MIC activity that is a similar concentration.

## 3. Discussion

β-lactam antibiotics have served their purpose for decades as a reliable and potent class of antibacterial drugs. However, increasing β-lactam resistance limits current treatment options severely, especially against infections caused by Gram-negative pathogens, such as *P. aeruginosa*. Many efforts have been directed towards the research and development of improved β-lactams or combinations of known β-lactams with novel β-lactamase inhibitors. Interestingly, two novel PBP inhibitors (e.g., boronate acids and DBOs) that inhibit the transpeptidation activity of PBPs and bypass β-lactam resistance were recently discovered and are currently in preclinical development [16,18,41,42]. Alternative approaches have been explored in the past to identify noncovalent PBP inhibitors. However, the potency of the identified compounds was disappointingly low [25,32,43,44,45,46]. Nevertheless, targeting the essential TPases remains of great interest and discovering novel entities capable of inhibiting these enzymes can contribute immensely to the fight against the antimicrobial resistance crisis.

In this work, we have optimised a high-throughput assay for screening compound libraries targeting *^Pa^*PBP3 and used this assay to identify a novel scaffold class of small molecules as *^Pa^*PBP3 inhibitors. The absorbance S2d assay approach has been traditionally used to investigate the TPase activities of several bacteria, but the assay has some drawbacks that limit its use as a target-based screening and identification of PBP inhibitors. Here, we provide a new fluorometric readout alternative that enabled us to eliminate interferences previously reported with screening compounds. The assay was used to screen a library of 2455 protease-targeted inhibitors. After eliminating compounds that did not display dose-dependent target inhibition or were considered as weak inhibitors, a set of nine hit compounds based on a pyrrolidine-2,3-dione core was identified. Fluorescence polarization experiments indicated that these compounds are competitive inhibitors and can displace Bocillin FL binding at the catalytic TPase site. A small-focused library of pyrrolidine-2,3-dione-based derivates was designed and synthesised to investigate the SAR of this cluster. Initial MIC testing against *P. aeruginosa* isolates in the presence of PMBN showed antibacterial activity of this pyrrolidine-2,3-diones class with no obvious cytotoxic effects. The results are therefore encouraging and provide an opportunity to explore this chemical class for antibacterial drug discovery. However, without a clear understanding of a distinct binding mode of the most active compounds at the active centre of *^Pa^*PBP3, the data presented here cannot rule out the possibility of a secondary off-target activity for this compound class.

**Figure 4:**
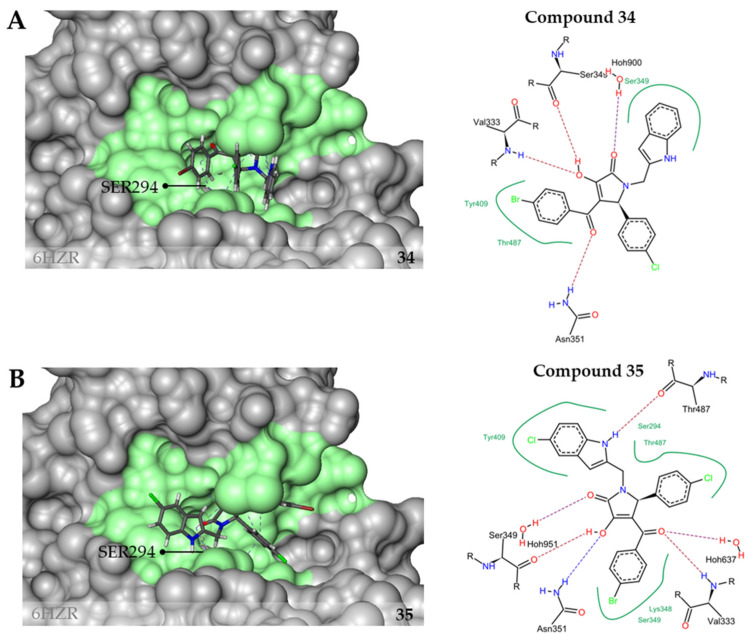
Docking model of pyrrolidine-2,3-dione compounds and *^Pa^*PBP3 (PDB ID:6HZR) [13]. (A) Best-fit position of compound **34**. (B) Best-fit position of compound **35**. Dot lines indicate hydrogen-bonds, green lines indicate potential hydrophobic interactions.

We generated an *in-silico* docking model (LeadIT version 2.3.2, BioSolveIT GmbH, Sankt Augustin, Germany) illustrating the potential binding mode of compounds **34** and **35** to *^Pa^*PBP3 (6HZR) [13]. Best-fit poses were defined using the LeadIT software when 50% of the maximum allowed overlap volume and 0.5 intra-ligand clashed. These simulations indicated that the essential hydroxyl group at position R^2^ interacts with the main-chain atoms of Val333 located at the TPase site (Figure 4). The secondary amine of the indole moiety and its connection to the pyrrolidone ring via a C2-linker in compounds **34** and **35** may facilitate an extra interaction with Thr487 that may explain the lower IC_50_ and *K*_d_ values of these compounds. These docking models are supported by the fluorescence polarisation experiments against Bocillin FL and strongly suggest that the compounds interact with the TPase domain. However, a cocrystal structure with *^Pa^*PBP3 would be required to confirm this hypothesis and provide a binding mode for further optimisation of the identified scaffold as potent PBP3 inhibitors. Moreover, further optimisation steps will focus on improving the physicochemical properties of the molecules. For example, reducing the hydrophobicity of the molecules may facilitate bacterial cell envelope penetration.

The work presented here underscores a novel class of PBP3 small molecule inhibitors, which offer excellent starting points for further hit-to-lead optimisation and development. The combination of promising *^Pa^*PBP3 inhibition, initial antibacterial activities and safe cytotoxic profile is highly encouraging. Further exploration of this chemical cluster will focus on implementing the “new rules of permeation” to advance this class of molecules to the periplasmic target for improved PBP3 inhibition and enforced antibacterial activity.

## 4. Materials and Methods

### 4.1. Chemicals

The SAR study of the pyrrolidine-2,3-dione cluster and the design of further analogons was performed at AiCuris. Custom synthesis of analogues and the S2d artificial substrate was subcontracted to Enamine Building Blocks Ltd. (Riga, LVA). The custom synthesised compounds were delivered as powder in amber–glass bottles and purities higher than 98%. Compound were stocked at 4 °C and 10 mM stock solutions in 96% DMSO were stored at 4 °C to avoid crystallisation.

*Representative synthesis of pyrrolidine-2,3-diones* [38,39]. A mixture of ethyl 4-(4-bromophenyl)-2,4-dioxobutanoate (0.125 g, 1.0 mmol) and *p*-chlorobenzaldehyde (0.14 g, 1.0 mmol) in dry dioxane (2 mL) was stirred for 30 min at 50 °C. Acetic acid (0.5 mL, 8.74 mmol) and *m*-fluoro-benzylamine (0.285 g, 1.0 mmol) were added. The resulting mixture was stirred for 2 h at 90 °C. After cooling the reaction mixture to r.t., the precipitate was filtered, washed with dioxane, and dried in vacuum to give 0.208 g of target compound **1** (0.415 mmol, 42% yield). ^1^H NMR (400 MHz, DMSO-*d_6_*): δ 7.65 (br, 4H), 7.32 (br, 5H), 7.06-7.09 (m, 1H), 6.89–6.95 (m, 2H), 5.33 (s, 1H), 4.75 (d, *J* = 12 Hz, 1H), 3.98 (d, *J* = 12 Hz, 1H); ^13^C NMR (100 MHz, DMSO-*d_6_*) δ 188.3, 165.9, 163.2 (d, ^1^*J*_CF_ = 139 Hz), 139.8, 139.8, 137.5, 135.4, 133.4, 133.3, 131.6, 131.1, 130.9, 130.9, 130.3, 129.0, 126.8, 124.1, 124.1, 119.2, 115.0, 114.8, 114.6, 114.5, 60.8, 44.2. APSI MS: M^+^+1 503.0.

*Analytical data of representative pyrroldine-2,3-diones*. 4-(4-bromobenzoyl)-5-(4-chlorophenyl)-3-hydroxy-1,5-dihydropyrrol-2-one (**32**). ^1^H NMR (400 MHz, DMSO-*d_6_*) δ 9.43 (s, 1H), 7.73–7.51 (m, 4H), 7.43–7.26 (m, 4H), 5.46 (s, 1H), 3.17 (br s, 1H); ^13^C NMR (100 MHz, DMSO-*d_6_*): δ 188.7, 167.4, 138.1, 137.6, 132.8, 131.7, 131.1, 129.6, 128.8, 126.8, 120.1, 56.6. HRMS (ESI): Calcl. mass C_17_H_11_BrClNO_3_ 390.9611 (M^+^), found 390.9609and 393.9663 (M^+^+1).

*4-(4-bromobenzoyl)-5-(4-chlorophenyl)-3-hydroxy-1-(1H-indol-2-ylmethyl)-5H-pyrrol-2-one* (**34**). ^1^H NMR (400 MHz, DMSO-*d_6_*): δ 12.23 (s, 1H), 11.03 (s, 1H), 7.64-748 (br, 4H), 7.46–7.36 (br m, 6H), 7.02 (br, 2H), 6.16 (s, 1H), 5.30 (s, 1H), 5.05 (d, *J* = 15.2 Hz, 1H), 3.88 (d, *J* = 15.7 Hz, 1H); ^13^C NMR (100 MHz, DMSO-*d_6_*): δ 188.2, 165.5, 137.5, 136.8, 135.4, 135.4, 134.1, 133.3, 131.6, 131.6, 131.1, 130.1, 130.1, 129.1, 128.2, 126.8, 121.5, 119.4, 111.6, 100.9, 60.3, 38.2. HRMS (ESI): Calc. mass C_26_H_18_BrClN_2_O_3_ 520.0189 [M^+^], found 520.0181 and 523.0234 (M^+^+1).

*4-(4-bromobenzoyl)-5-(4-chlorophenyl)-1-(furan-2-ylmethyl)-3-hydroxy-5H-pyrrol-2-one* (**41**). ^1^H NMR (400 MHz, DMSO-*d_6_*): δ 7.78–7.49 (br, 5H), 7.48–7.18 (br, 4H), 6.31 (s, 1H), 6.19 (s, 1H), 5.25 (s, 1H), 4.81 (d, *J* = 16.0 Hz, 1H), 3.89 (d, *J* = 16.1 Hz, 1H); ^13^C NMR (100 MHz, DMSO-*d_6_*): δ 189.2, 165.3, 149.7, 143.3, 137.4, 135.2, 135.2, 133.2, 131.6, 131.6, 130.0, 129.0, 126.9, 119.3, 110.9, 109.1, 60.5, 37.4. HRMS (ESI): Calc. mass C_22_H_15_BrClNO_4_ 470.9873 (M^+^), found 470.9873 and 473.9923 (M^+^+1).

4*-(4-bromobenzoyl)-5-(4-chlorophenyl)-3-(ethylamino)-1-[(3-fluorophenyl)methyl]-5H-pyrrol-2-one* (**43**). ^1^H NMR (400 MHz, DMSO-*d_6_*): δ 8.93 (br s, 1H), 7.45 (d, *J* = 8.0 Hz, 2H), 7.23–7.35 (m, 3H), 7.12 (d*, J* = 8.0 Hz, 1H), 7.06 (t, *J* = 8.0 Hz, 2H), 6.72–6.96 (m, 4H), 5.50 (s, 1H), 4.64 (d, *J* = 15.6 Hz, 1H), 3.83-3.88 (overlapping d with br s, 3H) 3.83-3.63 (br, 1H), 1.19 (t, *J* = 7.1 Hz, 3H); ^13^C NMR (100 MHz, DMSO-*d_6_*): δ 189.0, 162.6, 162.5 (d, ^1^*J*_CF_ = 194 Hz), 139.9, 139.8, 139.8, 136.3, 132.8, 131.5, 130.9, 130.8, 130.2, 130.1, 129.5, 128.6, 124.2, 124.2, 115.0, 114.8, 114.6, 114.5, 110.8, 62.0, 44.1. HRMS (ESI): Calc. mass C_26_H_21_BrClFN_2_O_2_ 526.0459 (M^+^), found 526.0453 and 529.0507 (M^+^+1).

*4-(4-bromobenzoyl)-5-(4-chlorophenyl)-1-[(3-fluorophenyl)methyl]-3-(morpholin-4-yl)-5H-pyrrol-2-one* (**44**). ^1^H NMR (400 MHz, DMSO-*d_6_*): δ 7.65 (d, *J* = 8.5 Hz, 2H), 7.55 (d, *J* = 8.4 Hz, 2H), 7.30 (q, *J* = 7.4 Hz, 1H), 7.23 (d, *J* = 8.4 Hz, 2H), 7.15 (d, *J* = 8.2 Hz, 2H), 7.06 (t, *J* = 8.3 Hz, 1H), 6.80 -6.92 (m, 2H), 5.25 (s, 1H), 4.64 (d, *J* = 15.4 Hz, 1H), 3.99 (d, *J* = 15.5 Hz, 1H), 3.46-3.52 (m, 6H), 2.93-3.02 (m, 2H); ^13^C NMR (100 MHz, DMSO-*d_6_*): δ 189.9, 166.6, 162.5 (d, ^1^*J*_CF_ = 197 Hz), 144.6, 138.6, 138.5, 138.3, 134.4, 134.1, 131.9, 130.4, 130.3, 129.7, 129.1, 129.0, 127.7, 124.1, 124.0, 118.6, 115.4 (d, ^2^*J*_CF_ = 17 Hz), 114.9 (d, ^2^*J*_CF_ = 17 Hz), 66.8, 61.9, 50.0, 44.2, 44.1. HRMS (ESI): Calc. mass C_28_H_23_BrClFN_2_O_3_ 568.0565 (M^+^), found 568.0557 and 571.0610 (M^+^+1).

### 4.2. Protein Expression and Purification

The soluble wild-type *P. aeruginosa* PBP3 (amino acids 40 to 563) was overexpressed using the plasmids pET41_PBP-3. The plasmid was transformed and expressed in the BL21 (DE3) Rosetta strain. The expression strain was inoculated from glycerol stock in two sequential LB medium precultures supplemented with kanamycin (50 µg/mL) and chloramphenicol (30 µg/mL) and incubated overnight at 37 °C and 160 rpm. Expression cultures were inoculated to OD_578_ 0.1 and were grown to the exponential phase in baffled flasks to improve aeration at 37 °C and 160 rpm. Induction of expression was done between OD_578_ 0.3 to 0.6 by the addiction of 1 mM isopropyl-β-D- thiogalactopyranoside (IPTG) and an incubation period of 20 h at 21 °C and 120 rpm. The cultures were then harvested (5000× *g*, 4 °C, and 15 min), washed with 50 mL of water and stored over night at −80 °C. Lysis of cell pellets was done via the Qproteome Bacterial Lysis Buffer (Qiagen, Hilden, Germany), following the manufacture’s protocol. Purification of the His-tagged PBP3 was done via immobilised metal affinity chromatography (IMAC) using the ÄKTA chromatography system (GE Heathcare, Freiburg, Germany) with HisTrap HP column (CV:2 mL). Washing and elution of the protein was done by imidazole gradients with a mix of IMAC Buffer A (1x Sorensen Buffer, 200 mM NaCl, 0.5 mM DDM, 10% glycerol, pH 7.5), and IMAC Buffer B (1× Sorensen Buffer (57 mM Na_2_HPO4 and 10 mM KH_2_PO_4_), 200 mM NaCl, 0.5 mM DDM, 10% glycerol, 300 mM Imidazole, pH 7.5) in 1 mL fractions up to 20× CV. The eluted protein solution was dialysed in 2 L of dialysis buffer (1× Sorensen Buffer, 200 mM, 20% glycerol, 0.5 mM DDM, pH 7.5). Protein concentrations were determined via photometric measurement.

### 4.3. IC_50_ Determination by S2d_fluo_

The activity of soluble *^Pa^*PBP3 (300 nM) was measured in the presence of 0.8 mM S2d, 1 mM D-alanine and 0.5 mM mBBr for 25 min by monitoring the increase in the fluorescence intensity of mBBr at Ex/Em: 394/490 nm using an Infinite M1000 microplate reader (Tecan, Männedorf, Switzerland). To test the effect of inhibitors, 100 µM or 0.8 to 100 µM of compounds diluted in 96% DMSO were incubated for 30 min and 450 rpm with 300 nM soluble PBP3. The incubation period was set to improve the binding of non-covalent inhibitors and 0.01% Triton X100 (Sigma-Aldrich, Taufkirchen, Germany) was added to prevent aggregation of compounds. The enzyme reaction was started by adding a substrate mix of 0.8 mM S2d and 0.05 mM mBBr. The enzyme, compound and substrate mix were mixed 1:1, pipetted with a Liquidator 96 (Melter Toledo, Gießen, Germany) and the fluorescence intensity was directly monitored with an Infinite M1000 microplate reader (Tecan) at Ex/Em: 394/490 nm. The assay was performed in black, flat-bottom 96-well microplates (Greiner Bio-One, Kremsmünster, Austria) at 37 °C in Sorensen Phosphate Buffer (57 mM Na_2_HPO_4_ and 10 mM KH_2_PO_4_, pH 7.5 + 0.01% Triton X-100). The velocity of the PBP3 reaction was calculated in the presence of each concentration of compound and the velocity values were used to estimate IC_50_ by sigmoidal nonlinear regression, using Prism v.9 software (San Diego, CA, USA) and the velocity of the reaction at each concentration.

### 4.4. IC_50_ Determination by the Bocillin FL Biochemical Assay

Fluorescence polarisation (FP) experiments were carried out as described before [32,35,36,47]. To determine the binding of Bocillin FL to PBP, 200 nM of *^Pa^*PBP3 was incubated for 30 min with 100 nM of Bocillin FL (Thermo Fisher Scientific, Waltham, MA, USA). The fluorescence polarisation signal of free Bocillin FL was monitored in a Pherastar microplate reader (BMG Labtech, Ortenberg, Germany) in the polarisation mode with Ex/Em: 485/520 nm excitation and emission filters. Millipolarisation (mP) units were calculated using the MARS data analysis software v30.40.R2 (BMG Labtech, Ortenberg, Germany). All the experiments were done in Sorensen buffer at 37 °C using flat-bottom, black 96-well plates. To determine the IC_50_ values of the potential Bocillin FL competitors, 5 µL of serial compound dilutions (100 µM to 0.8 µM compound) in 96% DMSO were incubated with PBP3 of *P. aeruginosa* for 30 min to promote binding of the compounds. Bocillin FL was diluted in Sorensen buffer and added to the reaction and directly measured for 30 min at 37 °C. The FP was continuously measured in the presence and absence of compound and enzyme. The differences between the mP values at 0 min and 17 min were used as the parameter to estimate the IC_50_ values. The estimated IC_50_ values were established by sigmoidal nonlinear regression, using Prims v.8 software.

### 4.5. SPR Experiments 

SPR experiments were performed at 25 °C by using a Biacore T200 instrument from Cytiva (Marlborough, MA, USA). Purified *^Pa^*PBP3 was coated on a CM5 chip, that was activated with EDC/NHS (1-ethyl-3-(3-dimethylaminopropyl) carbodiimide/N-hydroxysuccinimide) beforehand and blocked with ethanolamine after protein attachment. We achieved a protein amount of 11.820 RU. For every analyte, two concentration series between 3.25 µM and 200 µM were prepared in the running buffer (13 mM NaCl, 257 µM KCl, 171 µM KH_2_PO_4_, 950 µM Na_2_HPO_4_, 0.05% Tween-20 and 5% DMSO, pH 7.4) in a 96 well plate. Prior to the injection, the baseline was determined using running buffer for 300 s. Then, the analytes were then associated for 100 s and dissociated for 300 s with a flow rate of 30 µL/min. A 30 s washing step with 50% DMSO was used after every injection to regenerate the chip. In the final step, ten different DMSO concentrations ranging from 4.0% to 5.8% were used as the internal solvent correction. Steady state analysis was performed by using the internal Biacore software and figures were prepared with Prism v.8 using the points of one concentration series, respectively.

### 4.6. Bacterial Strains

The wild-type *P.aeruginosa* PAO1 and the isogenic *P. aeruginosa* K2896 Δ*mexB*Δ*mexXY*Δ*mexCD*-*oprJ* isolates were used to determine the MIC in the presence and absence of PMBN [48].

### 4.7. MIC Determination

MIC determination was performed via standard broth microdilution according to the Clinical & Laboratory Standards institute guidelines [49,50]. Overnight colonies grown on BD-Columbia agar with 5% Sheep Blood (Becton Dickinson, Franklin Lakes, NJ, USA), were diluted in Mueller Hinton Broth 2 cation adjusted (MH+) medium (Thermo Fisher Scientific) to a concentration of 5 × 10^5^ CFU/mL. Serial dilutions (1:2) of test compounds were incubated at 37 °C with 5 × 10^4^ bacteria in a final volume of 100 μL. Sterile and growth controls in the presence of 1% DMSO and PMBN if the case, and the positive controls, aztreonam (AZT) and tigecycline (TIG) were included in the experiments (Table S4). The MICs were determined visually after 18 h at 37 °C and confirmed by the addition of 10% PrestoBlue (ThermoFisher Scientific) reagent according to the manufacture’s protocol. The fluorescence signal of PrestoBlue reagent was measured at Ex/Em: 550/595 nm after 30 min incubation at 37 °C using at a ClarioStar microplate reader (BMG Labtech). PrestoBlue reduction occurs in the presence of living cells and consequently turns red in colour and is highly fluorescent.

### 4.8. Cytotoxicity Assays

Cell lines stored at −120 °C were thawed to 37 °C and resuspended in 10 mL of medium pre-warmed to 37 °C. Table S6 summarises the culture media and conditions for each cell line. Cells were pelleted at 300× *g* for 10 min, and then resuspended in fresh medium, seeded in a T-75 culture flask, and placed in a CO_2_ incubator (37 °C with 5% CO_2_ tension). Once the cells reached approximately 70–80% confluency, they were then passaged regularly. The adherent cell lines NRK52E, HepG2 and Huh 7 were washed once with 10 mL DPBS and detached with Trypsin-EDTA for 5 to 7 min and resuspended in 15 mL of the appropriate medium. The suspension cell line CCRF-CEM were diluted and resuspended in fresh RPMI 1640 PAN medium. To calculate the cell density, the cell resuspensions were diluted 1:6 with DPBS. Equal volumes of cell suspension and Trypan Blue were mixed and gently loaded into a haemocytometer (INCYTO, 31056 Republic of Korea). In each experiment, cell concentrations were adjusted to reach a concentration of 2.5 × 10^3^ cell/well for NRK52E, 5 × 10^3^ cell/well for HepG2, 6.2 × 10^4^ cell/well for CCRF-CEM and 3 × 10^3^ for H9. The cells were incubated with decreasing concentration of compounds (1 µL in 96% DMSO) for 72 h at 37 °C with 5% CO_2_ tension. Cycloheximide, a cytotoxic compound that interferes with the translocation step in eukaryotic protein synthesis was used as a control for the experiments. The assay was carried out in flat-bottom, transparent 96-well plates. Cell viability was determined using the PrestoBlue reagent (Thermo Fisher Scientific, Waltham, MA, USA) according to the manufacture’s protocol. First, 10× PrestoBlue was added to each well to obtain a 1× solution, and incubated at 37 °C for 45 min. Then, fluorescence was measured at Ex/Em: 550/595 nm using a ClarioStar microplate reader (BMG Labtech). Cytotoxicity was determined as the percentage of the difference in absorbance of the untreated sample to the treated sample. CC_50_ was calculated using the values obtained in each concentration using the sigmoidal dose-response by Prism v.8. The media used in each cell line is detailed in Table S6.

## Figures and Tables

**Table 1:** Biochemical characterisation of the pyrrolidine-2,3-dione cluster.

				IC_50_ Determination with *^Pa^*PBP3 ^a^ (µM)	
Compound		Source	PBP3 Inhibition at 100 µM (%)	S2d_fluo_ Assay	FP-Bocillin FL Assay
**1**	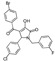	MPI ^b^	ND ^b,c^	28 ± 9	7 ± 3
**2**	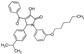	ChemDiv 8015-0105	101 ± 0.4	4 ± 6	17 ± 9
**3**	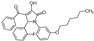	ChemDiv 8015-0104	100 ± 0.8	19 ±1	>100
**4**	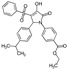	ChemDiv F232-0415	101	24 ± 20	>100
**5**	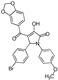	MPI	ND ^b,c^	61 ± 1	69 ± 13
**6**	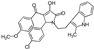	ChemDiv 8015-3001	92 ± 7	63 ± 4	9 ± 6
**7**	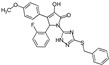	ChemDiv C202-3660	62	65 ± 19	58 ± 21
**8**	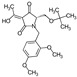	MPI [33]	ND ^b,c^	66 ± 16	93 ± 18
**9**	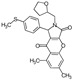	ChemDiv D159-0900	60	99 ± 1	18 ± 3

^a^ The IC_50_ values represent the average and the standard deviation of three independent measurements; ^b^ Max Plank Institute of Molecular Physiology, Department of Chemical Biology, Dortmund, Germany; ^c^ ND, not determined.

**Table 2:** Characterisation of the pyrrolidine-2,3-dione cluster. The biochemical characterisation of the pyrrolidine-2,3-dione cluster was determined by S2d_fluo_, fluorescence polarisation (FP) with Bocillin FL and surface plasmon resonance (SPR). The data represent the average and the standard deviations of three independent replicates. The minimal inhibitory concentration (MIC) against two *P. aeruginosa* strains was determined in the presence of PMBN. The cytotoxicity of the compounds was determined against the T-lymphoblast CCRF-CEM cell line, and the average and standard deviation from three independent measurements is indicated.

	Biochemical Characterisation ^a^			MIC (µM) against *P. aeruginosa*		Cytotoxicity CCRF-CEM (µM)
Nr	IC_50_ (µM) S2d_fluo_ Assay	IC_50_ (µM) FP-Assay	*K*_d_ (µM) SPR	PAO1 *^b^*	K28926 *^c^*	
**1**	28 ± 9	7 ± 3	ND	>100	12.5	>100
**32**	>100	>100	ND	>100	>100	94 ± 6
**33**	>100	>100	ND	>100	>100	58 ± 6
**34**	14 ± 9	4 ± 4	6.44	3.13	12.5	>100
**35**	3 ± 1	0.6± 2	9.82	3.13	12.5	53 ± 5
**36**	>100	>100	ND	>100	100	1 ± 2
**37**	16 ± 8	17 ± 3	454	6.25	12.5	8 ± 6
**38**	100 ± 11	117 ± 4	62.7	>100	>100	>100
**39**	24 ± 7	11 ± 4	ND	6.25	25	>100
**40**	8 ± 4	4 ± 2	22.9	25	>100	93 ± 4
**41**	>100	>100	ND	>100	25	>100
**42**	>100	>100	ND	>100	>100	>100
**43**	>100	>100	ND	>100	>100	73 ± 9.2
**44**	>100	>100	ND	>100	>100	52 ± 3
**45**	>100	>100	ND	>100	>100	23 ± 11

^a^ PBP3 of *P. aeruginosa*; *^b^* PAO1 (wild-type strain) with 4 µg/mL PMBN; *^c^* K28926 (ΔmexB; ΔmexX, ΔmexCD-oprJ) with 4 µg/mL PMBN; FP, fluorescence polarisation; SPR, surface plasmon resonance; ND: not determined.

## Data Availability

The data presented in this study are available on request from the corresponding author.

## Supplementary Materials

The following are available online at https://www.mdpi.com/article/10.3390/antibiotics10050529/s1, Table S1. Additional HTS statistical parameters; Table S2. Pyrrolidine-2,3-dione screening hits without concentration-dependent inhibition; Table S3. Minimal Inhibitory Concentrations of pyrrolidine-2,3-dione against wild-type strains; Table S4. MIC of control compounds against selected strains Table S5. Detailed cytotoxicity of pyrrolidine-2,3-dione against several cell lines; Table S6. Eukaryotic cell and culture media; Figure S1. NMR spectra of representative pyrrolidine-2,3-diones.
