# Supplementary material for: Discovery of Pyrrolidine-2,3-diones as Novel Inhibitors of P. aeruginosa PBP3
Source: Antibiotics (Basel). 2021 May 4;10(5):529. doi: 10.3390/antibiotics10050529 (PMC8147781; doi:10.3390/antibiotics10050529)
Supplement: Supplementary file 1 [file antibiotics-10-00529-s001.zip › antibiotics-1206078-supplementary.pdf]

SUPPLEMENTARY DATA

Table S1.

Table S1. High Throughput Screening validation by statistical parameters

	V_{PBP3} ± SD	V_{S2d} ± SD	S/N	Z'factor	CV_{PBP3} ± SD	CV_{S2d} ± SD
HTS assay	8.81 ± 0.68 (*n* = 378)	1.29 ± 0.19 (*n* = 378)	41.77	0.70	0.60 ± 0.3	0.98 ± 0.34

V_{PBP3}: Mean of Positive Control (PC) was defined as the activity of ^{PA}PBP3 in presence of D-alanine; V_{S2d}: Mean of Negative Control was defined as the S2d spontaneous hydrolysis; SD: Standard Deviations were calculated from the data obtained; S/N: Signal-to-Noise ratio indicates the difference between the PC and NC; an excellent assay should have a Z' factor between 0.5 and 1; CV: Coefficient of Variation of each signal is acceptable if < 20%.

Table S2

Table S2. Pyrrolidine 2,3-dione screening hits without concentration-dependent inhibition

		**Biochemical inhibition of ^{Pa}PBP3**	
**Compound**	**Source**	**Inhibition at 100 μM compound (%)**	**IC_{50} (μM) S2d-TP Assay**
10	ChemDiv F455-0278	60 ± 21	>100
11	ChemDiv F455-0164	86 ± 7	>100
12	ChemDiv F455-0912	76 ± 32	>100
13	ChemDiv F455-0267	85 ± 7	>100
14	ChemDiv F455-0279	70 ± 19	>100
15	ChemDiv F455-0467	71 ± 12	>100
16	ChemDiv F455-0396	65 ± 11	>100
17	ChemDiv F455-0454	95 ± 4	>100
18	ChemDiv	70 ± 21	>100
19	ChemDiv F455-0609	98 ± 4	>100
20	ChemDiv F455-0624	71 ± 13	>100
21	ChemDiv F455-0762	78 ± 31	>100
22	ChemDiv F455-0588	88 ± 14	>100
23	ChemDiv F455-0758	78 ± 9	>100
24	ChemDiv F455-0187	66 ± 7	>100
25	ChemDiv F455-0644	70 ± 18	>100
26	ChemDiv F455-1012	87 ± **32**	>100
27	ChemDiv F455-1004	78 ± 30	>100
28	ChemDiv R095-0004	57 ± 57	>100
29	ChemDiv F455-1003	84 ± 27	>100
30	ChemDiv F455-1013	87 ± 22	>100

Table S3

Table S3. Minimal Inhibitory Concentrations of pyrrolidine-2,3-dione against wild-type strains

	**MIC (µM) against different strains**							
	P. aeruginosa[a]		*E. coli*	*K. pneumoniae*	*A. baumanii*	*S. pneumoniae*	*B. subtilis*	*S. aureus*
	PAO1	K2896	Neumann	*clinical isolate*	ATCC15308 [b]		168	133
**1**	>100	12.5	>100	>100	>100	25	>100	>100
**32**	>100	>100	>100	>100	>100	>100	100	>100
**33**	>100	>100	>100	>100	>100	50	>100	>100
**34**	3.13	12.5	>100	>100	>100	12.5	25	6.25
**35**	3.13	12.5	>100	>100	>100	6.25	6.25	3.13
**36**	>100	100	>100	>100	>100	>100	>100	>100
**37**	6.25	12.5	>100	>100	>100	50	3.13	6.25
**38**	>100	>100	>100	>100	>100	25	>100	>100
**39**	6.25	25	>100	>100	>100	25	>100	25
**40**	25	>100	>100	>100	>100	12.5	>100	>100
**41**	>100	25	>100	>100	>100	50	>100	>100
**42**	>100	>100	>100	>100	>100	>100	>100	>100
**43**	>100	>100	>100	6.25–3.13	>100	>100	>100	>100
**44**	>100	>100	>100	>100	>100	>100	>100	>100
**45**	>100	>100	>100	>100	>100	25	>100	6.25

[a] *4 µg/mL PMBN,* [b] *10 µg/mL PMBN*

TABLE S4

Table S4. MIC of control compounds against selected strains.

		MIC (µM) against selected bacteria[1]	
Strain Name	PMBN (µg/ mL)	Aztreonam	Tygecyclin
P. aeruginosa PAO1	4	0.58	0.4
	0	>1.2	>4
P. aeruginosa K2896	4	0.29	0.2
	0	0.575	0.8
E. coli Newmann	0	0.287	0.4
A. baumannii ATCC15308	50	0.58	0.4
	0	>1.2	0.9
K. pneumoniae clinical isolate	50	0.071	0.4
	0	0.143	0.9
S. pneumoniae	0	>1.2	0.1
B. subtilis 168	0	>1.2	0.2
S. aureus 133	0	>1.2	0.2

[1] Values represent the median of at least three independent experiments

Table S5

Table S5. Detailed cytotoxicity of pyrrolidine-2,3-dione against four eukaryotic cell lines

Compound	Cytotoxicity effect (µM)			
	**Epithelial liver HepG2**	**Epithelial kidney NRK52E**	**T-lymphoblast CCRF-CEM**	**T-lymphocyte H9**
**1**	>100	>100	>100	>100
**32**	>100	98 ± 5	94 ± 6	>100
**33**	>100	84 ± 5	58 ± 6	>100
**34**	>100	>100	>100	>100
**35**	>100	53 ± 5	>100	96 ± 7
**36**	1 ± 2	58 ± 3	9 ± 4	48 ± 2
**37**	20 ± 4	9 ± 12	8 ± 6	19 ± 6
**38**	>100	>100	>100	>100
**39**	>100	>100	>100	>100
**40**	>100	>100	93 ± 4	>100
**41**	>100	>100	>100	>100
**42**	>100	>100	>100	>100
**43**	>100	73 ± 9.2	>100	>100
**44**	>100	52 ± 3	>100	>100
**45**	23 ± 8	23 ± 11	52 ± 8	**32** ± 10
**Cycloheximide**	1.34 ± 0.7	0.20 ± 0.2	0.66 ± 0.5	**0.53** ± 0.3

Table S6

Table S6. Eukaryotic cell and culture media

Cell line	Media composition
CCRF-CEM ATCC® CCL-119™	**RPMI 1640 PAN**<br>L-Glutamine<br>2,0 g/L $NaHCO_3$, 10% FCS, 1% L-Glutamine, 1% Pen/Strep
Huh 7<br>(Prof. Bartenschlager, Heidelberg University)	**DMEM PAN**<br>+ 4,5 g/L Glucose, L-Glutamine, Pyruvate, 3,7 g/L $NaHCO_3$, 10% FCS, 1% L-Glutamine, 1% Pen/Strep, 1% NEAA
HepG2 ATCC® HB-8065™	**MEM PAN**<br>+ 4,5 g/L Glucose, L-Glutamine, Pyruvate, 3,7 g/L $NaHCO_3$, 10% FCS, 1% L-Glutamine, 1% Pen/Strep, 1% NEAA
NRK52E ATCC® CRL-1571™	**DMEM PAN**<br>+ 4,5 g/L Glucose, L-Glutamine, Pyruvate, 3,7 g/L $NaHCO_3$, 10 % FCS, 1% Na-Pyruvate, 1% L-Glutamine, 1 % Pen/Strep

Figure S1: NMR Spectra of representative pyrroldine-2,3-diones:

Compound 1.

pdm3262.fid

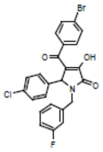

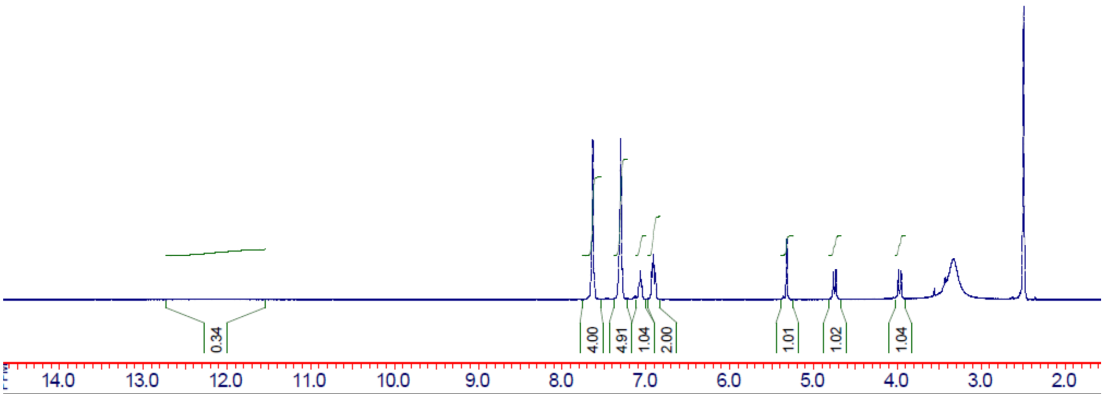

File name: pdm3262.fid	Operator:	SF: 499.8279 MHz	NSC: 0	PW: 9.80 usec, RG: 12
Date: 16-May-2019	Solvent: dmso	SW: 8993 Hz	TE: 323 K	AQ: 1.78 sec, RD: 0.00 sec

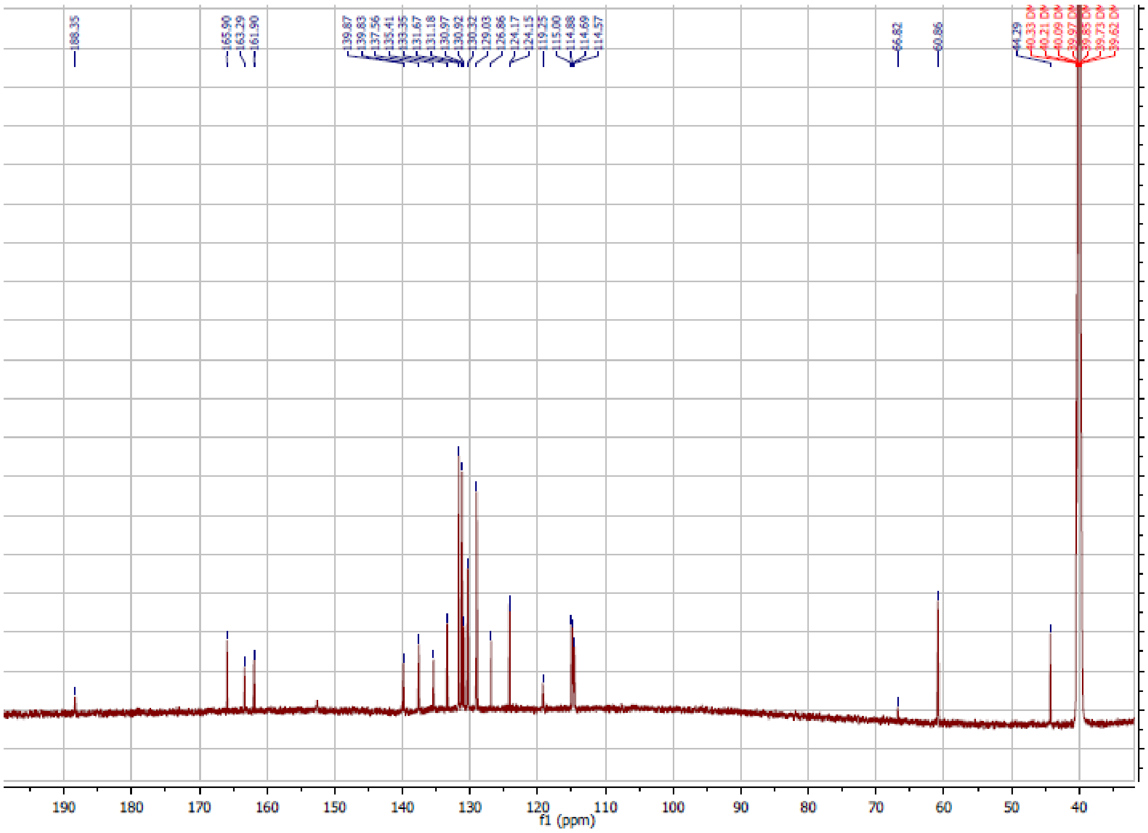

Compound 32.

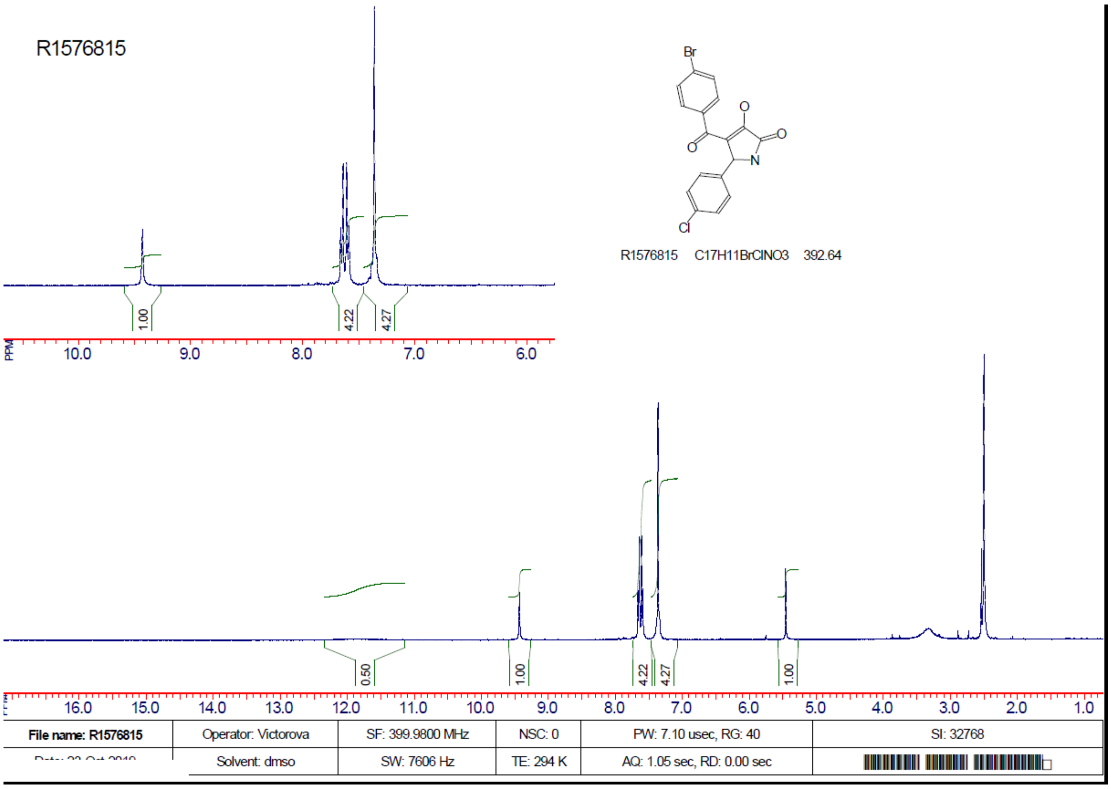

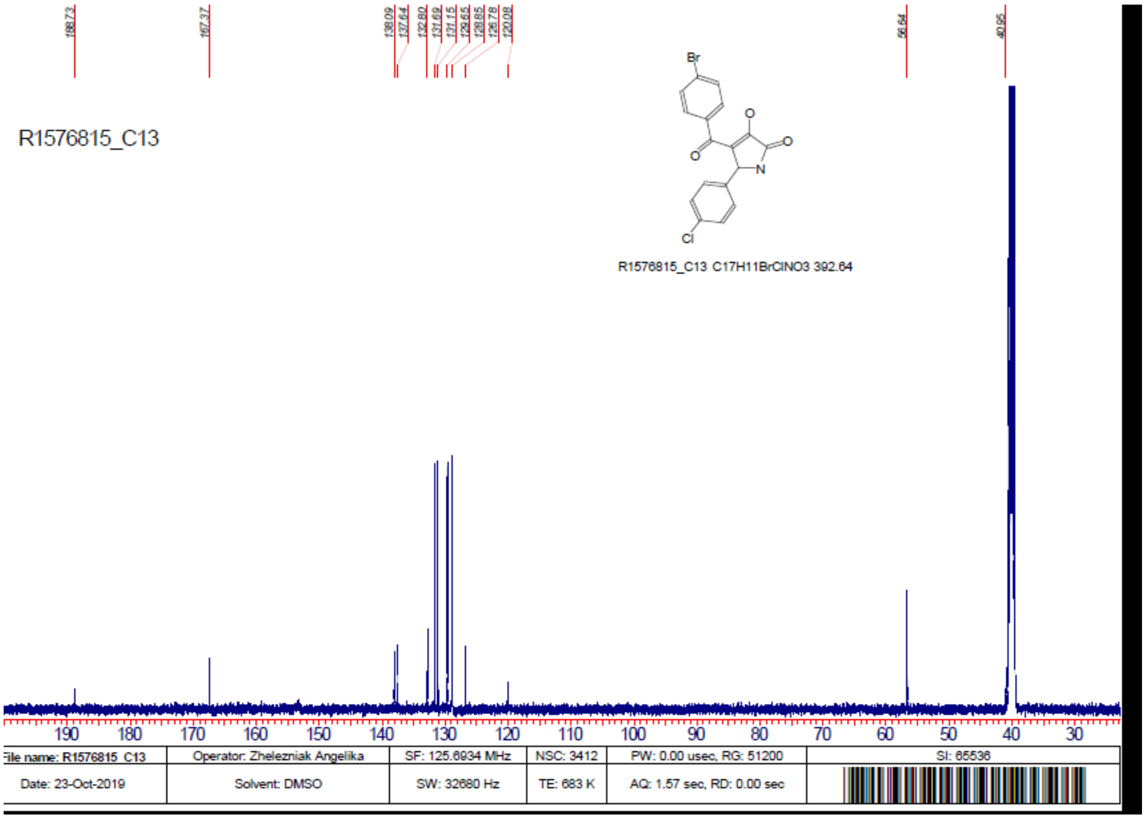

Compound 33.

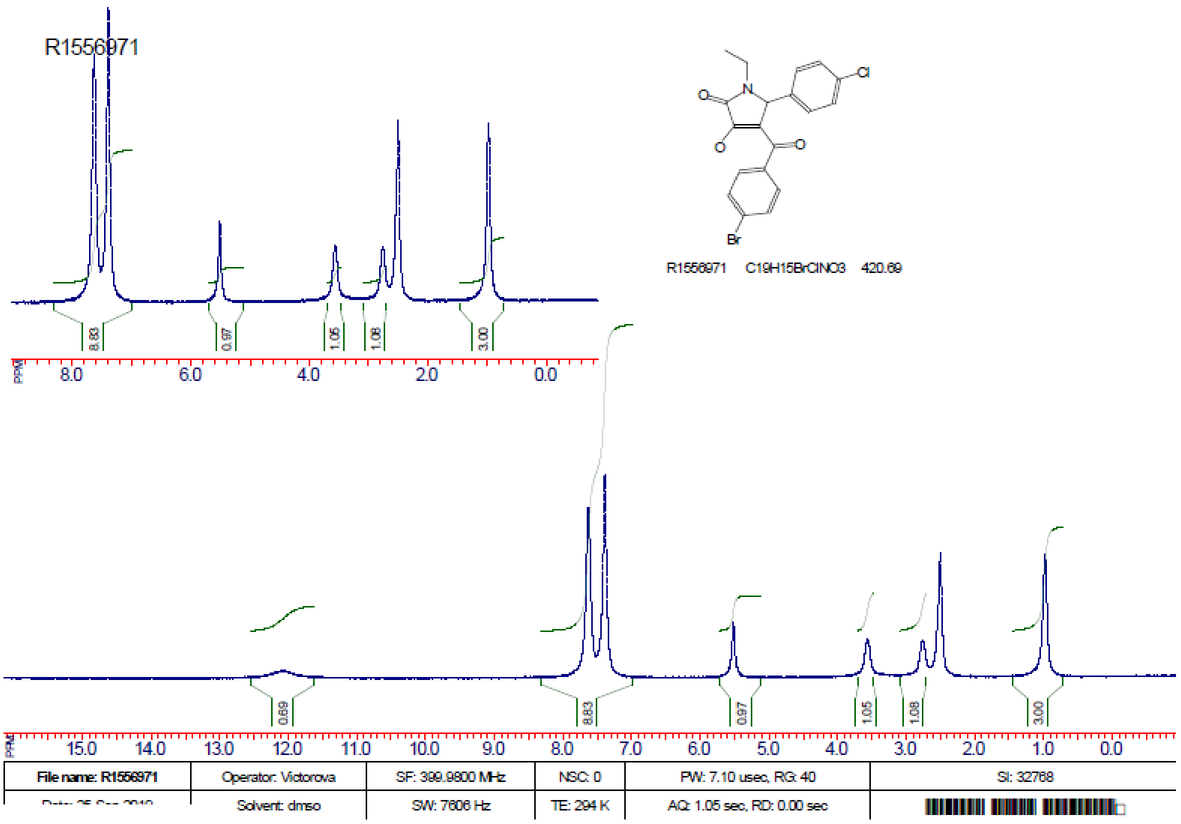
R1556971
R1556971 C19H15BrClNO3 420.69
File name: R1556971
Operator: Victorova
SF: 399.9800 MHz
NSC: 0
PW: 7.10 usec, RG: 40
SI: 32768
Solvent: dmso
SW: 7606 Hz
TE: 294 K
AQ: 1.05 sec, RD: 0.00 sec

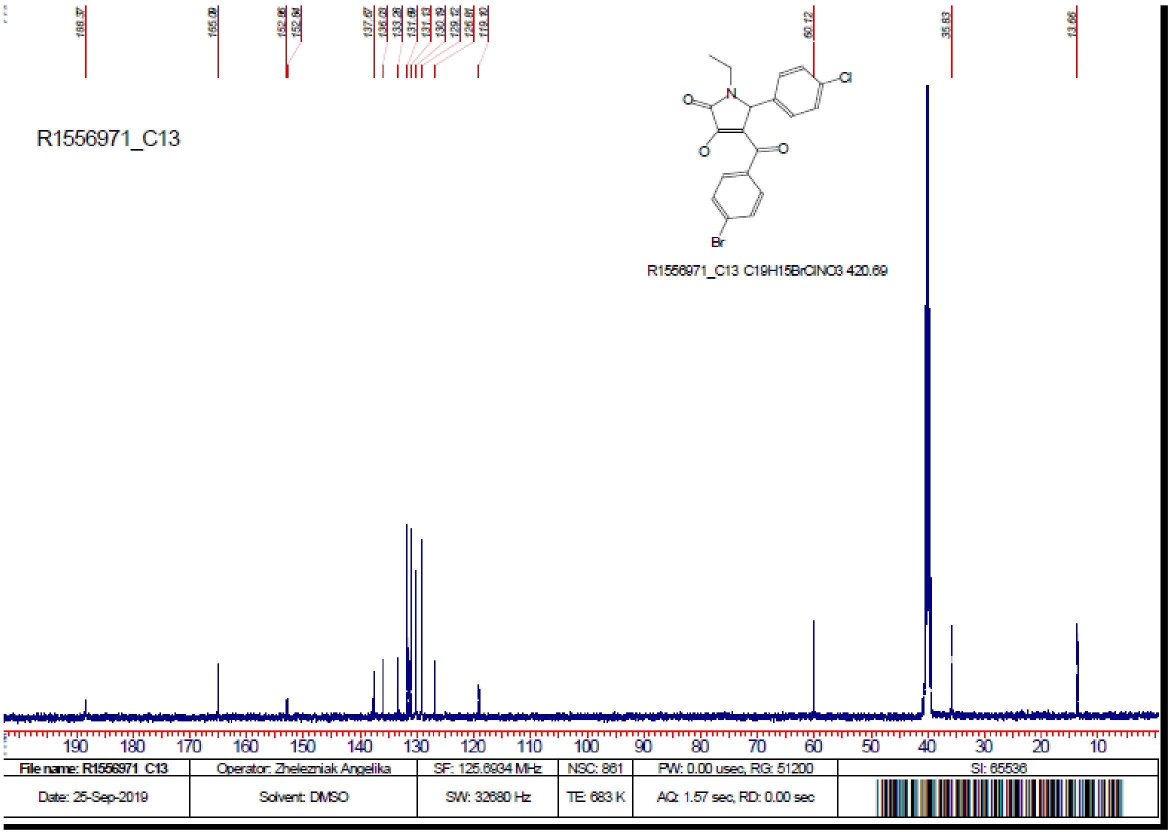
R1556971_C13
R1556971_C13 C19H15BrClNO3 420.69
File name: R1556971_C13
Operator: Zhelezniak Angelika
SF: 125.6934 MHz
NSC: 861
PW: 0.00 usec, RG: 51200
SI: 65536
Date: 25-Sep-2019
Solvent: DMSO
SW: 32680 Hz
TE: 683 K
AQ: 1.57 sec, RD: 0.00 sec

Compound 34

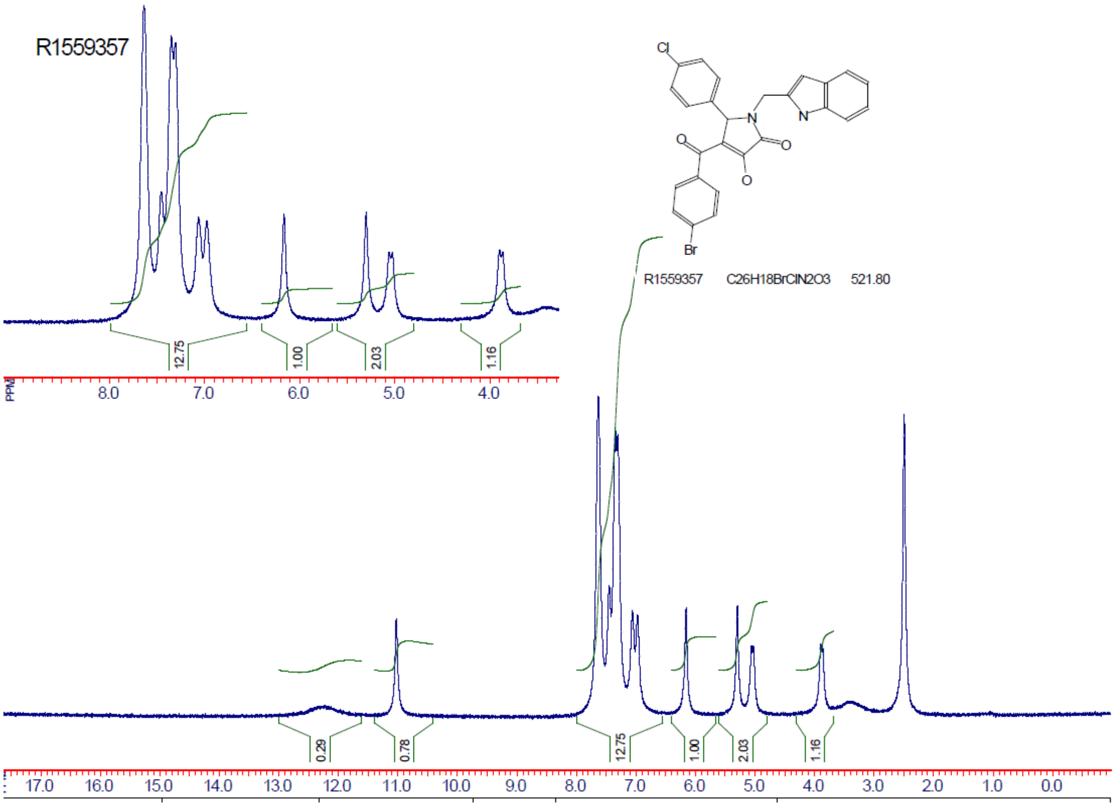
R1559357
Cl
N
N
O
O
O
Br
R1559357 C26H18BrClN2O3 521.80
12.75
1.00
2.03
1.16
0.29
0.78
PPM

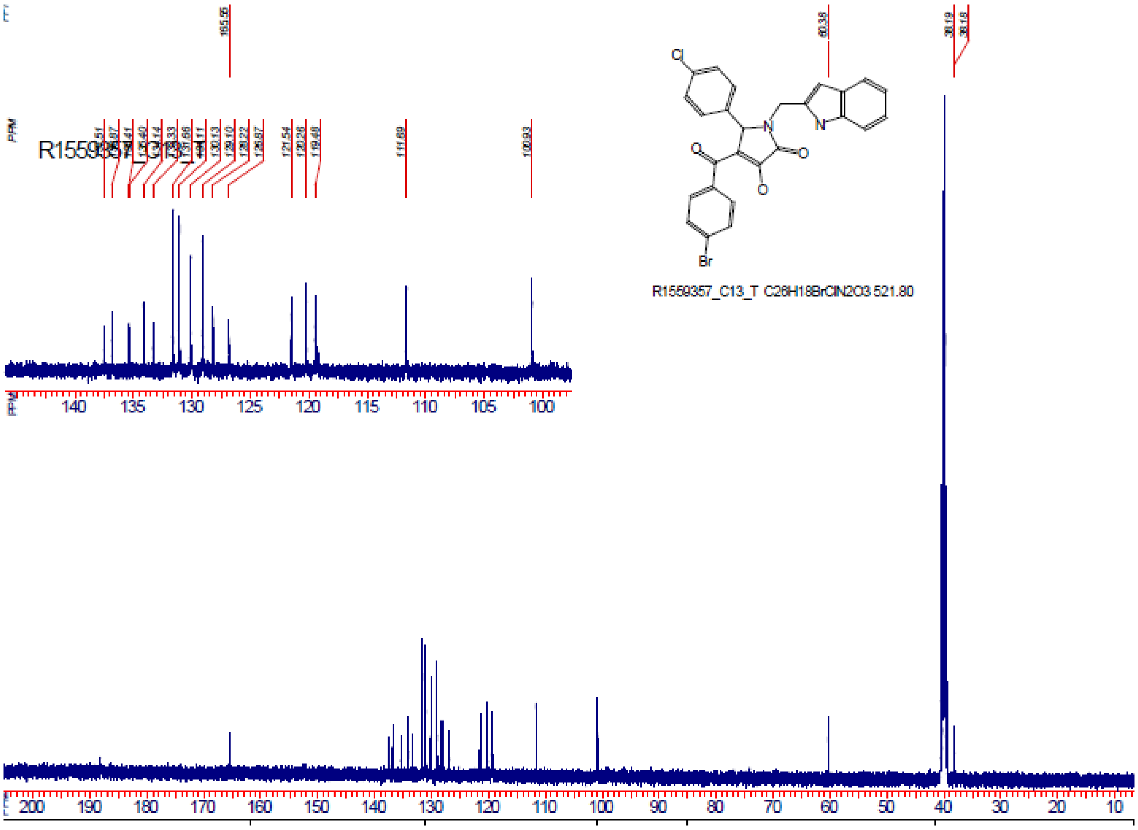
Cl
N
N
O
O
O
Br
R1559357_C13_T C26H18BrClN2O3 521.80
165.95
60.38
PPM

Compound 35.

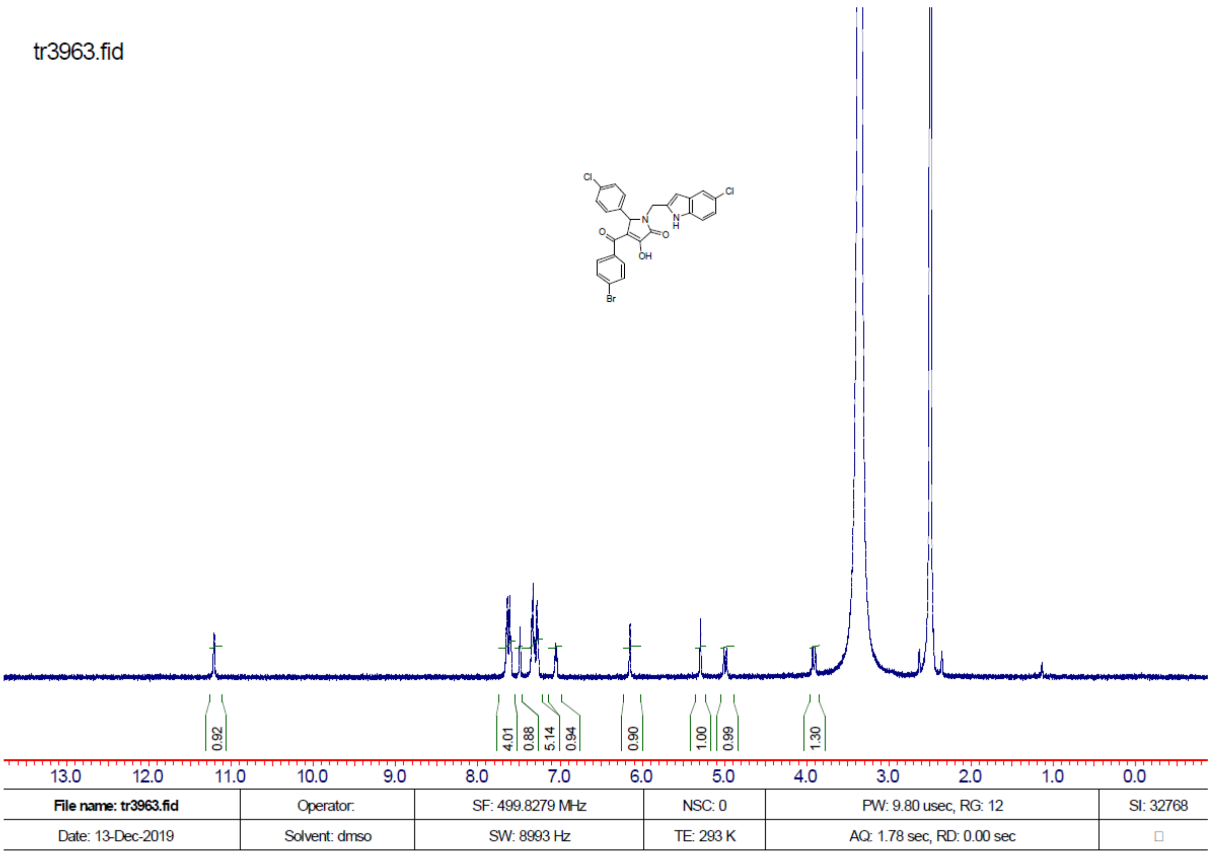
tr3963.fid
File name: tr3963.fid
Date: 13-Dec-2019
Operator:
Solvent: dmso
SF: 499.8279 MHz
SW: 8993 Hz
NSC: 0
TE: 293 K
PW: 9.80 usec, RG: 12
AQ: 1.78 sec, RD: 0.00 sec
SI: 32768

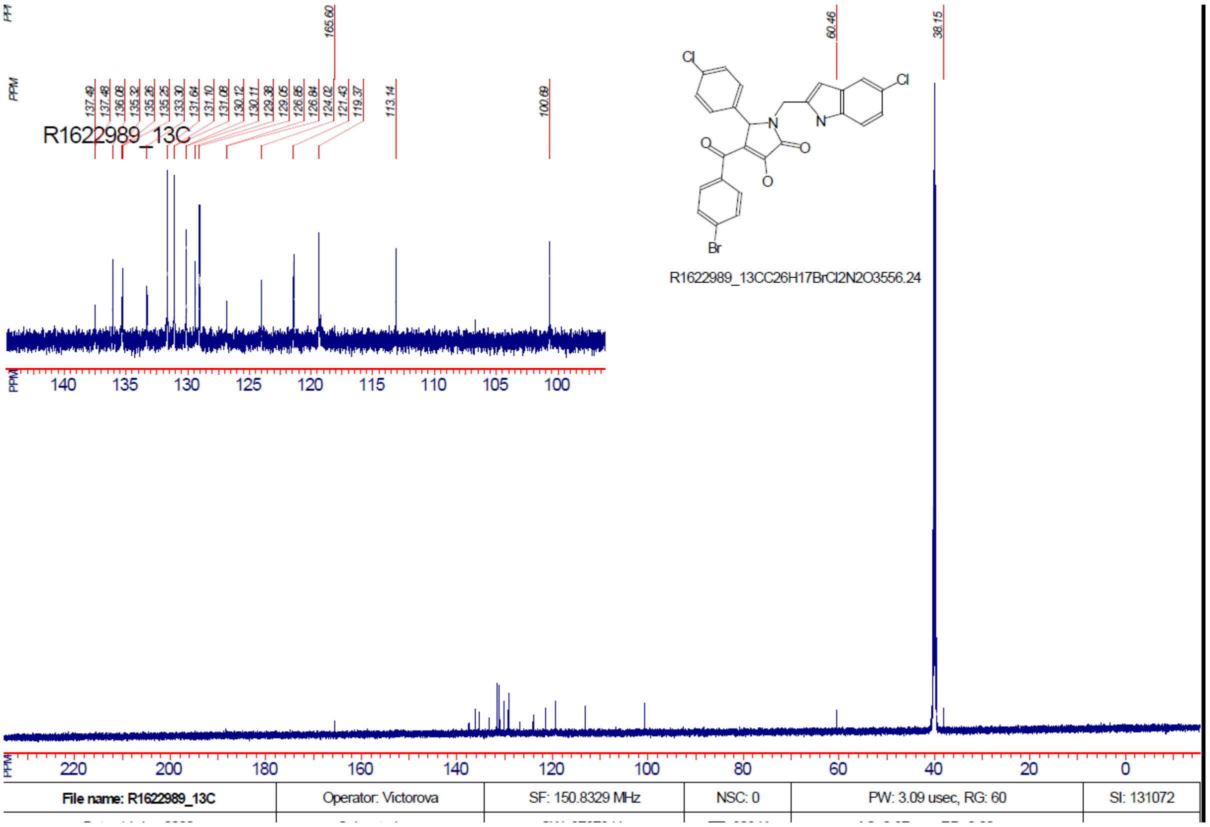
R1622989_13C
R1622989_13CC26H17BrCl2N2O3556.24
165.60
60.46
38.15
137.49
137.48
136.08
135.32
135.26
135.25
133.30
131.64
131.10
131.08
130.12
130.11
129.38
129.05
126.85
126.84
124.02
121.43
119.37
113.14
100.69
File name: R1622989_13C
Operator: Victorova
SF: 150.8329 MHz
NSC: 0
PW: 3.09 usec, RG: 60
SI: 131072

Compound 36.

^{1}H NMR spectrum of **KK05-06**:

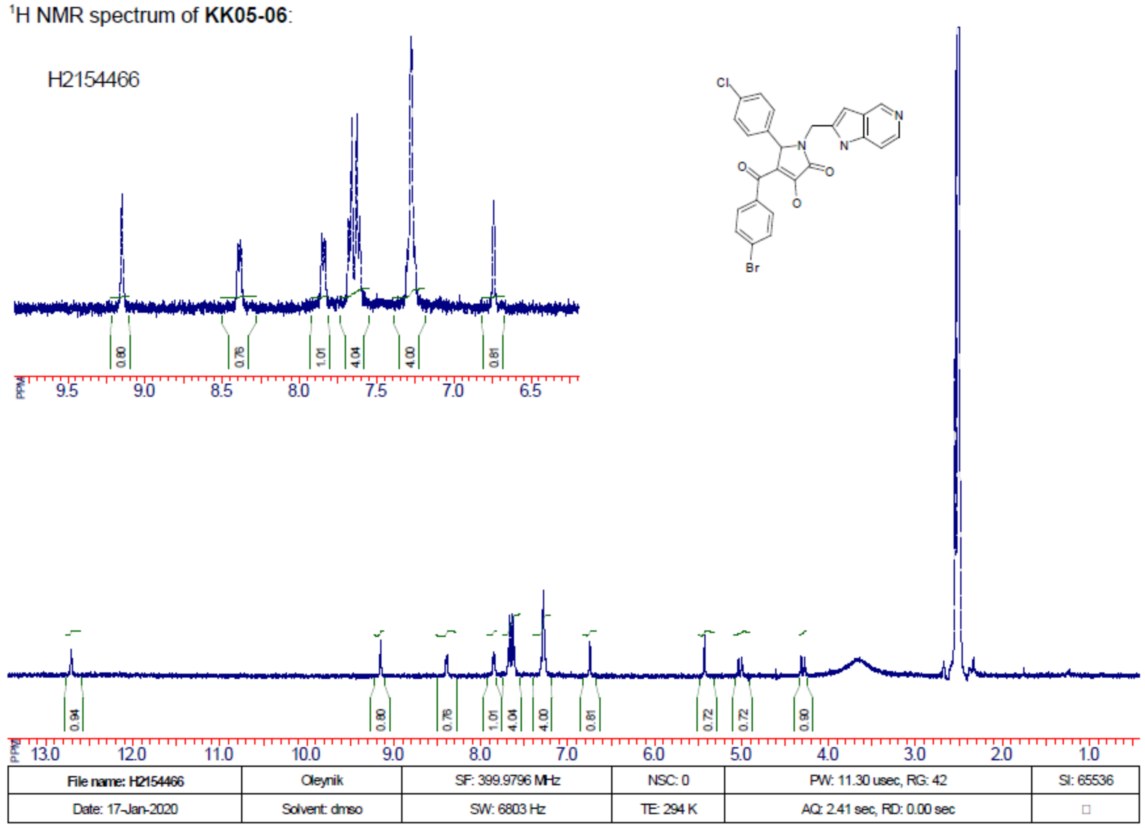

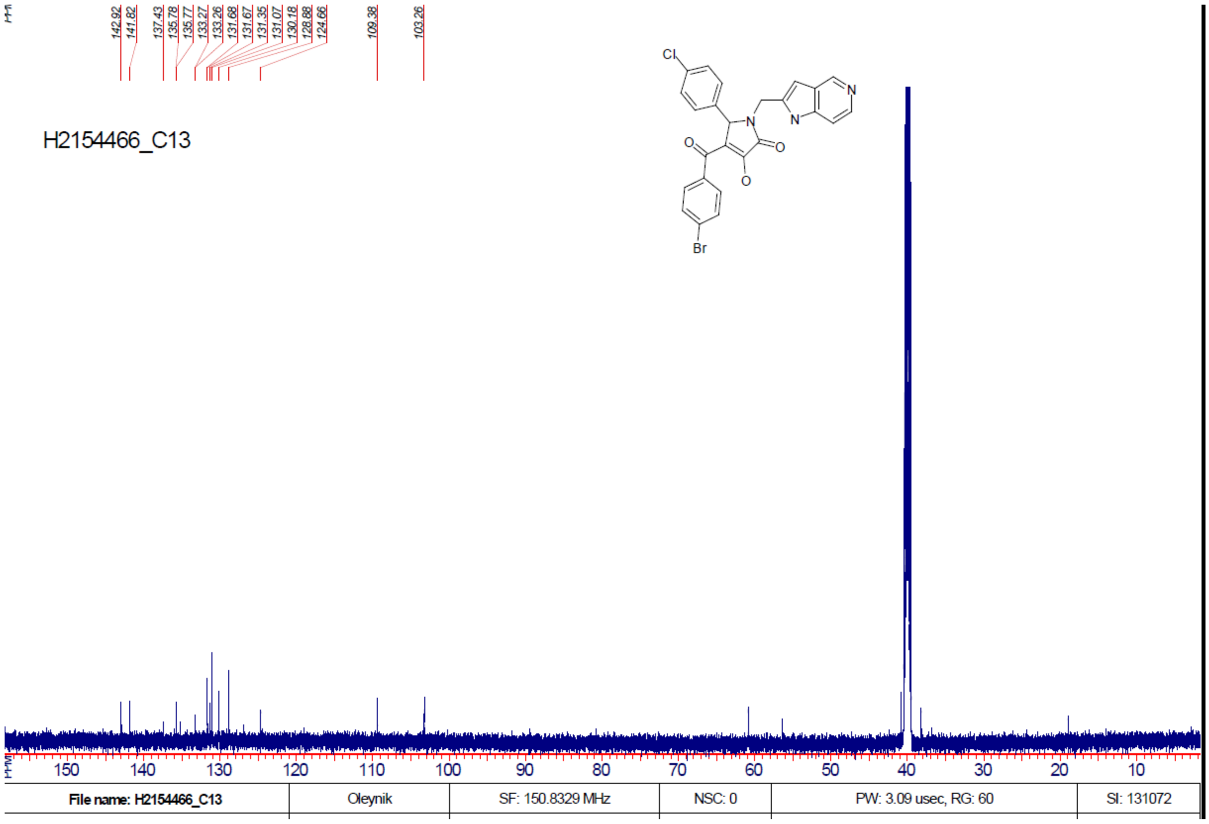

Compound 37.

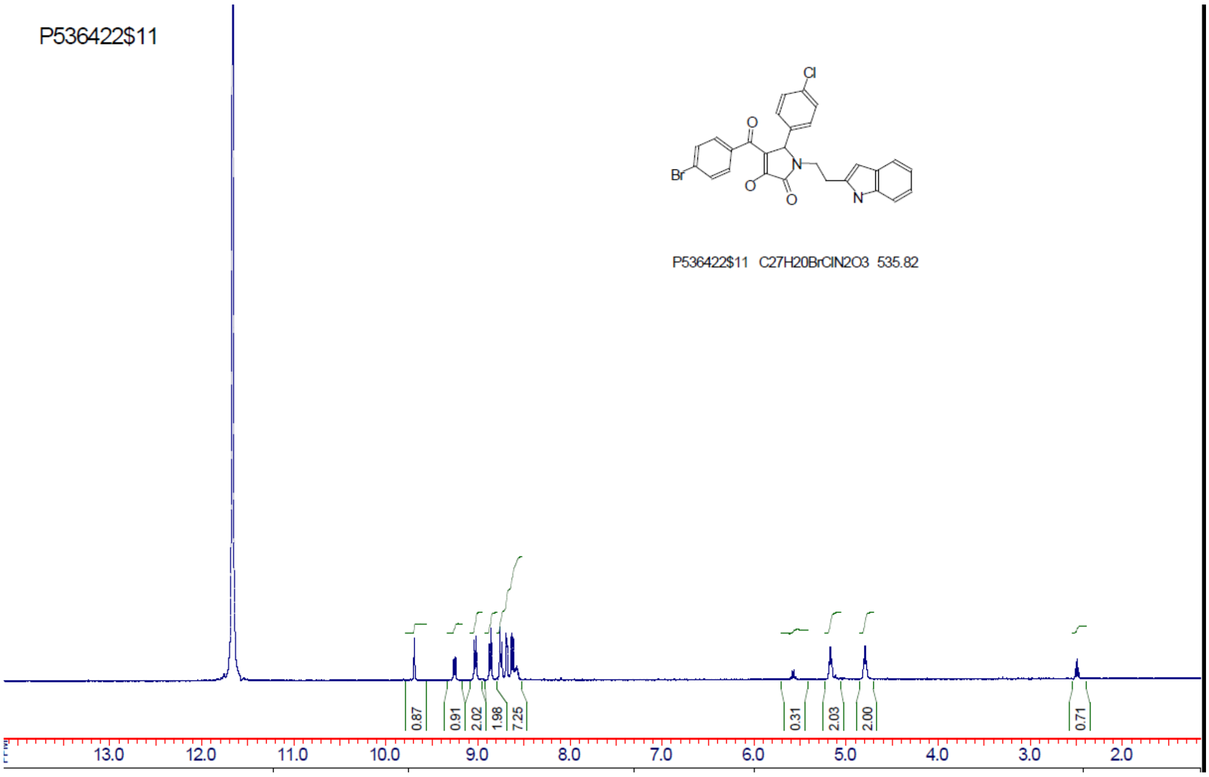
P536422$11
P536422$11 C27H20BrClN2O3 535.82
0.87
0.91
2.02
1.98
7.25
0.31
2.03
2.00
0.71
13.0
12.0
11.0
10.0
9.0
8.0
7.0
6.0
5.0
4.0
3.0
2.0

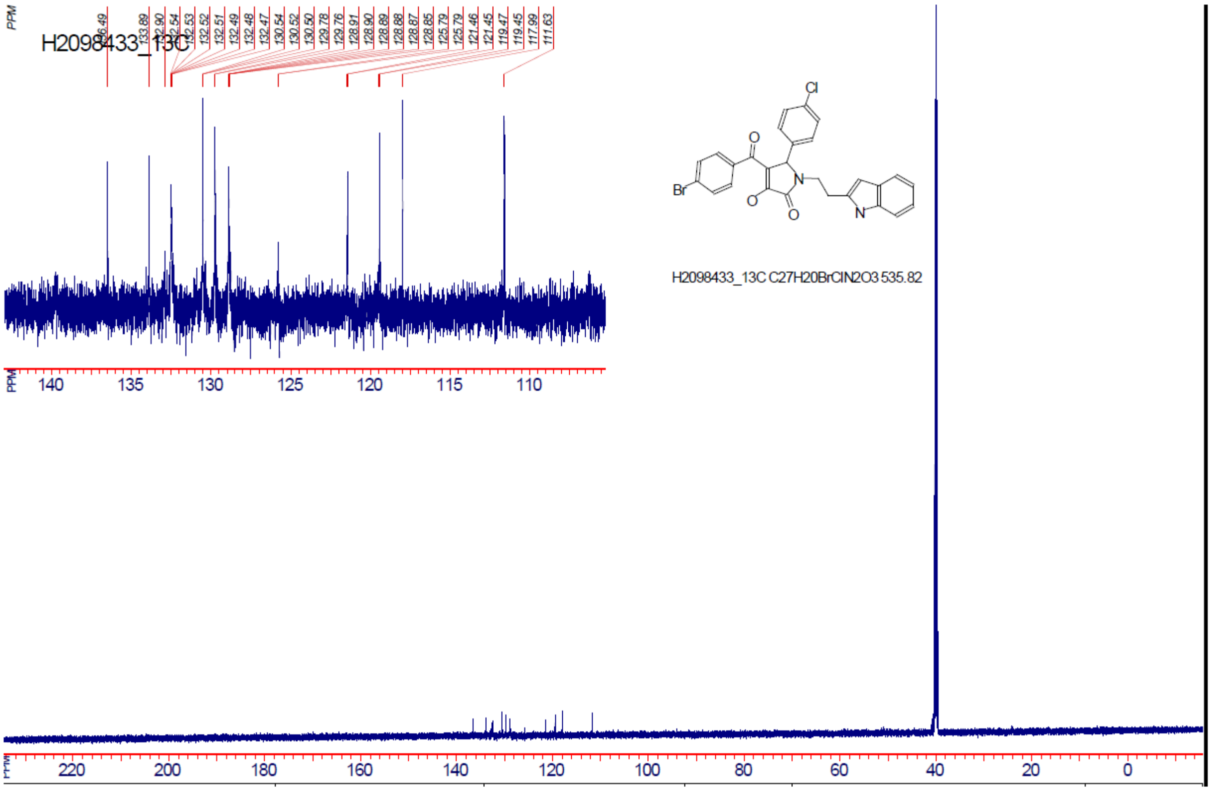
H2098433_13C
H2098433_13C C27H20BrClN2O3 535.82
PPM
140
135
130
125
120
115
110
220
200
180
160
140
120
100
80
60
40
20
0

Compound 38.

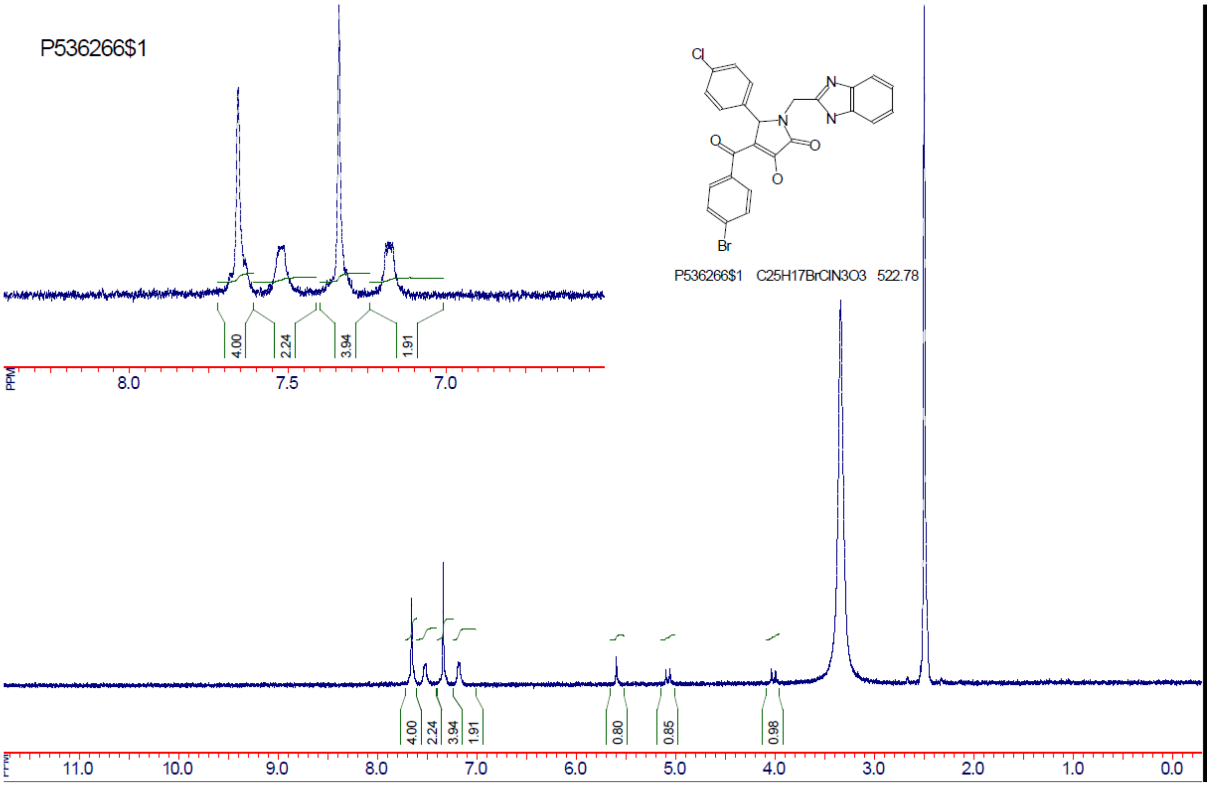
P536266$1
P536266$1 C25H17BrClN3O3 522.78
Cl
Br
N
O
4.00
2.24
3.94
1.91
0.80
0.85
0.98
PPM

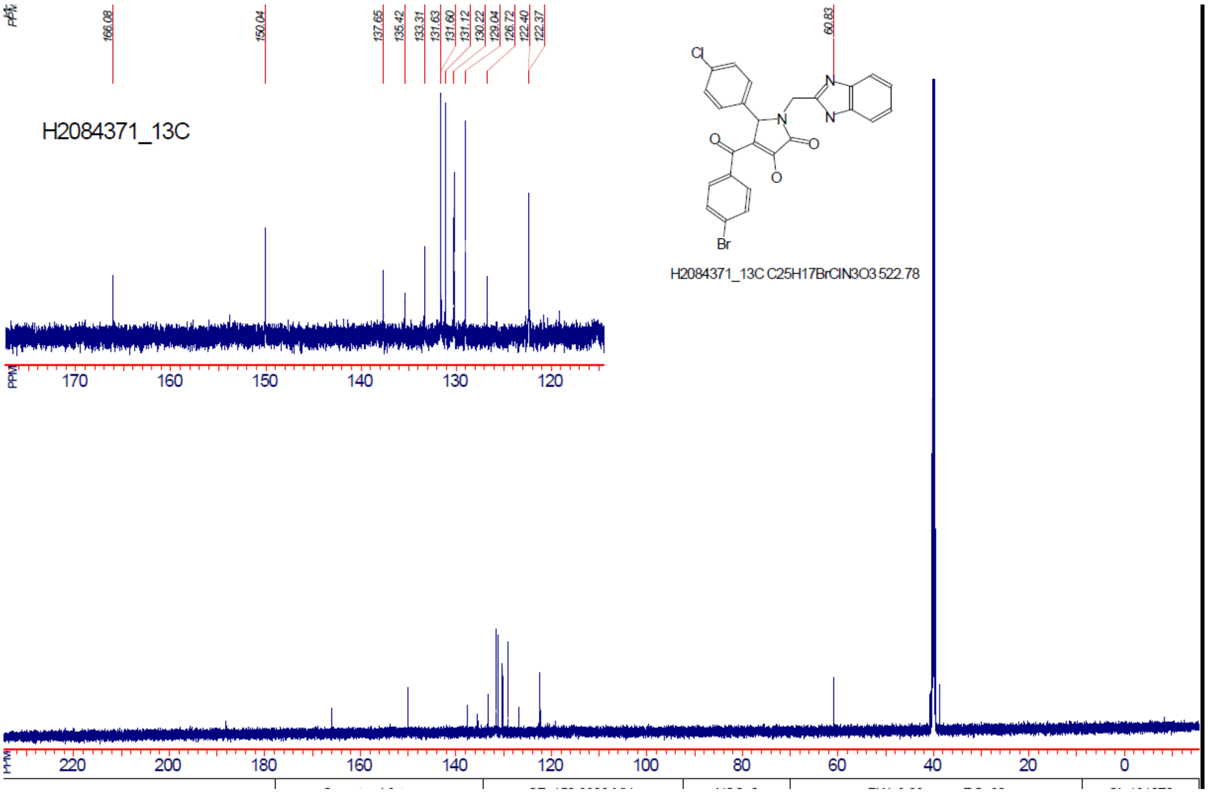
H2084371_13C
H2084371_13C C25H17BrClN3O3 522.78
Cl
Br
N
O
166.08
150.04
137.65
135.42
133.31
131.63
131.60
131.12
130.22
129.04
126.72
122.40
122.37
60.83
PPM

Compound 39.

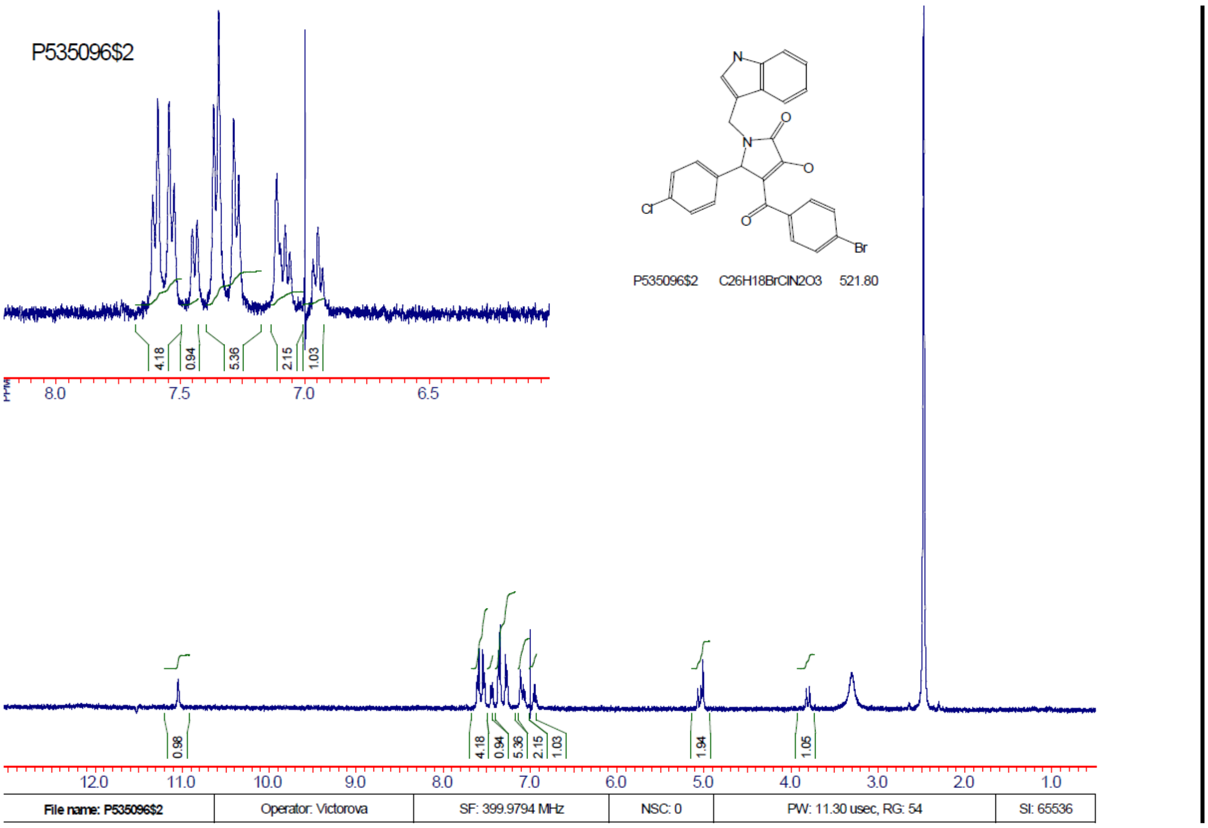
P535096$2
P535096$2 C26H18BrClN2O3 521.80
4.18
0.94
5.36
2.15
1.03
8.0
7.5
7.0
6.5
0.98
1.94
1.05
12.0
11.0
10.0
9.0
8.0
7.0
6.0
5.0
4.0
3.0
2.0
1.0
File name: P535096$2
Operator: Victorova
SF: 399.9794 MHz
NSC: 0
PW: 11.30 usec, RG: 54
SI: 65536

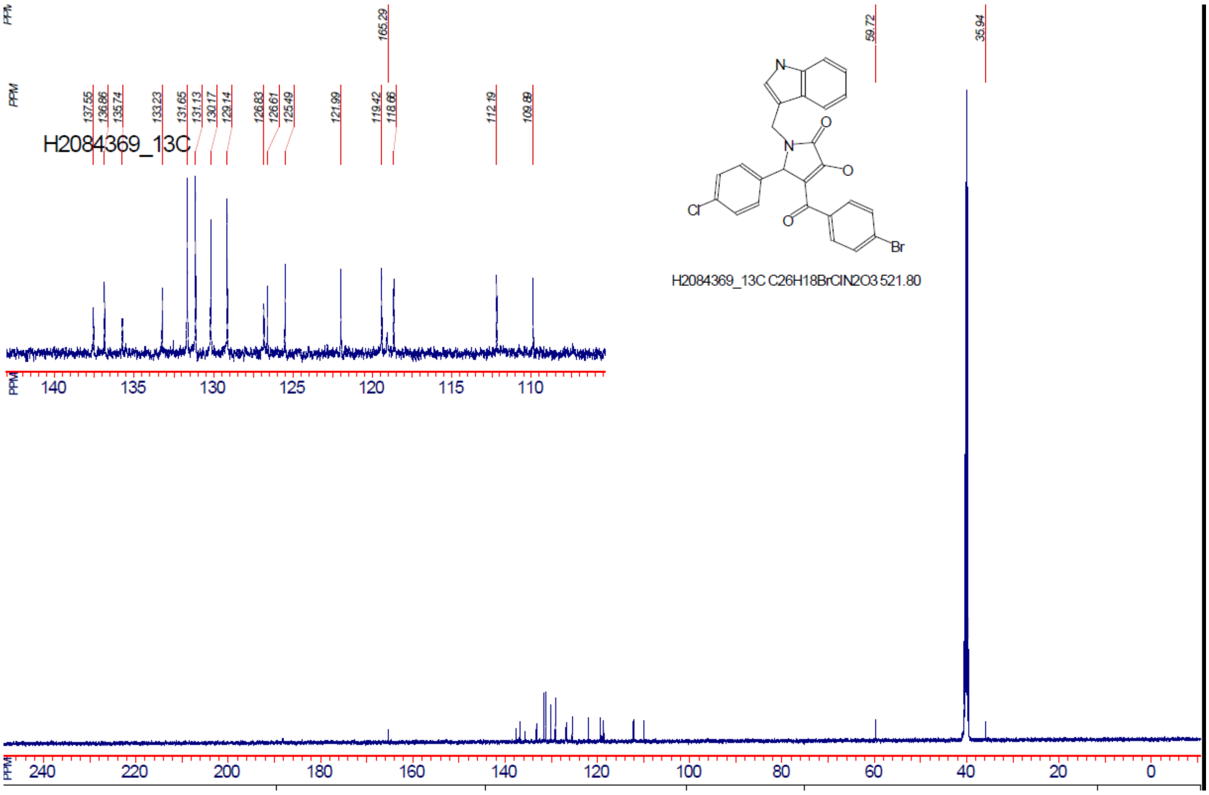
H2084369_13C
H2084369_13C C26H18BrClN2O3 521.80
165.29
59.72
35.94
137.55
136.86
135.74
133.23
131.65
131.13
130.17
129.14
126.83
126.61
125.49
121.99
119.42
118.66
112.19
109.89
140
135
130
125
120
115
110
240
220
200
180
160
140
120
100
80
60
40
20
0

Compound 40.

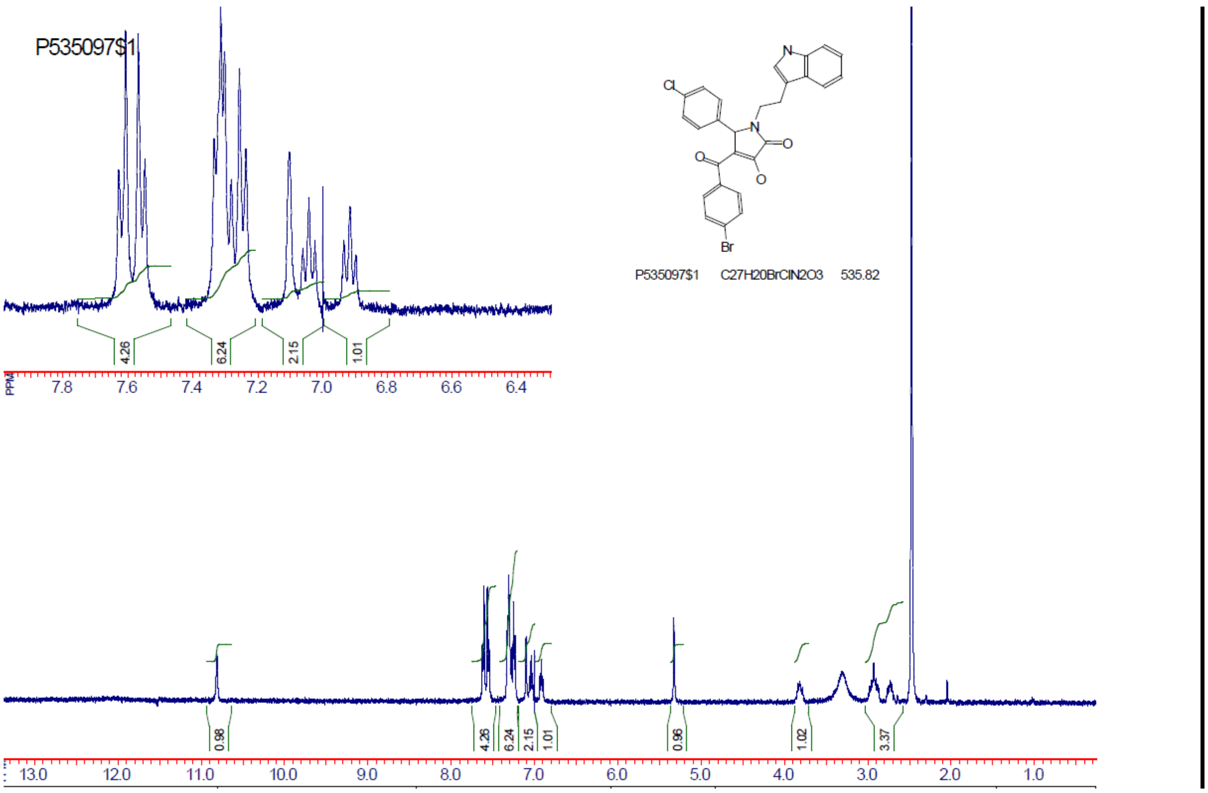
P535097$1
P535097$1 C27H20BrClN2O3 535.82
4.26
6.24
2.15
1.01
0.98
0.96
1.02
3.37

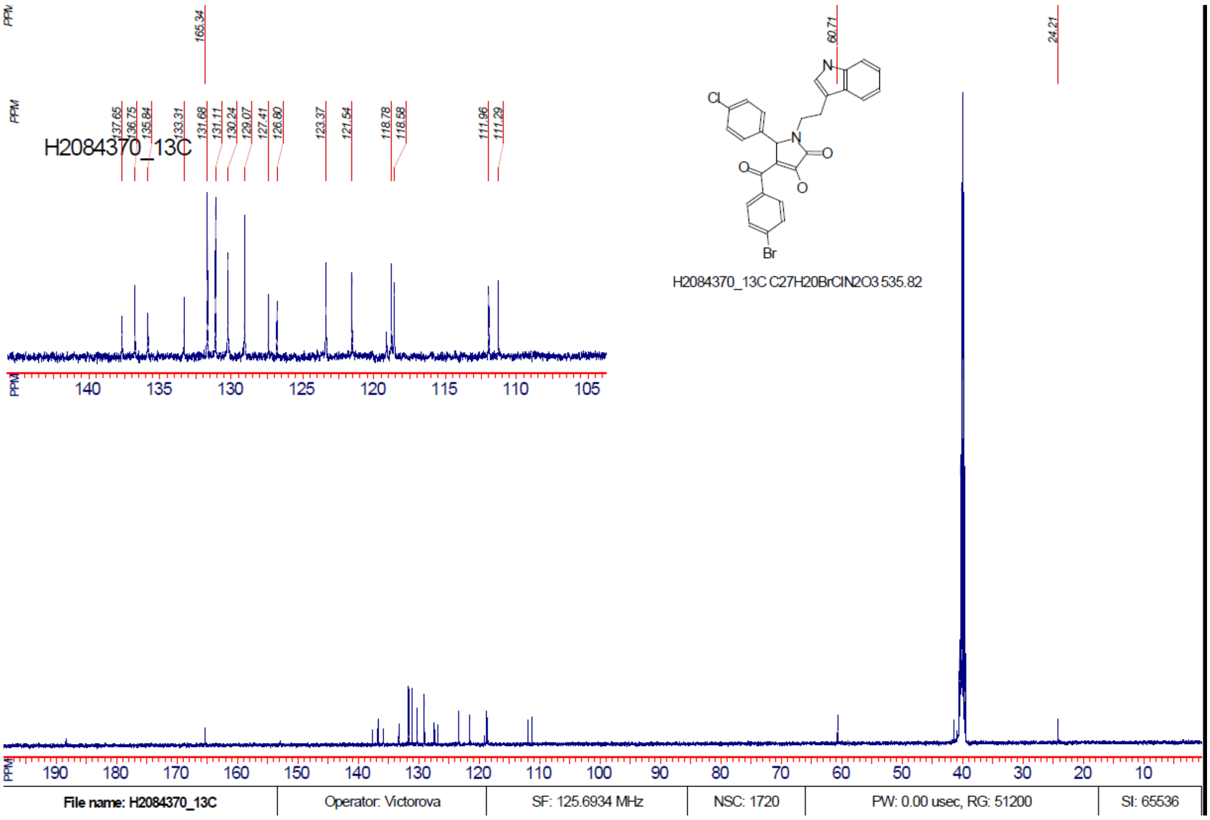
H2084370_13C
H2084370_13C C27H20BrClN2O3 535.82
165.34
60.71
24.21
137.65
136.75
135.84
133.31
131.68
131.11
130.24
129.07
127.41
126.80
123.37
121.54
118.78
118.58
111.96
111.29
File name: H2084370_13C
Operator: Victorova
SF: 125.6934 MHz
NSC: 1720
PW: 0.00 usec, RG: 51200
SI: 65536

Compound 41.

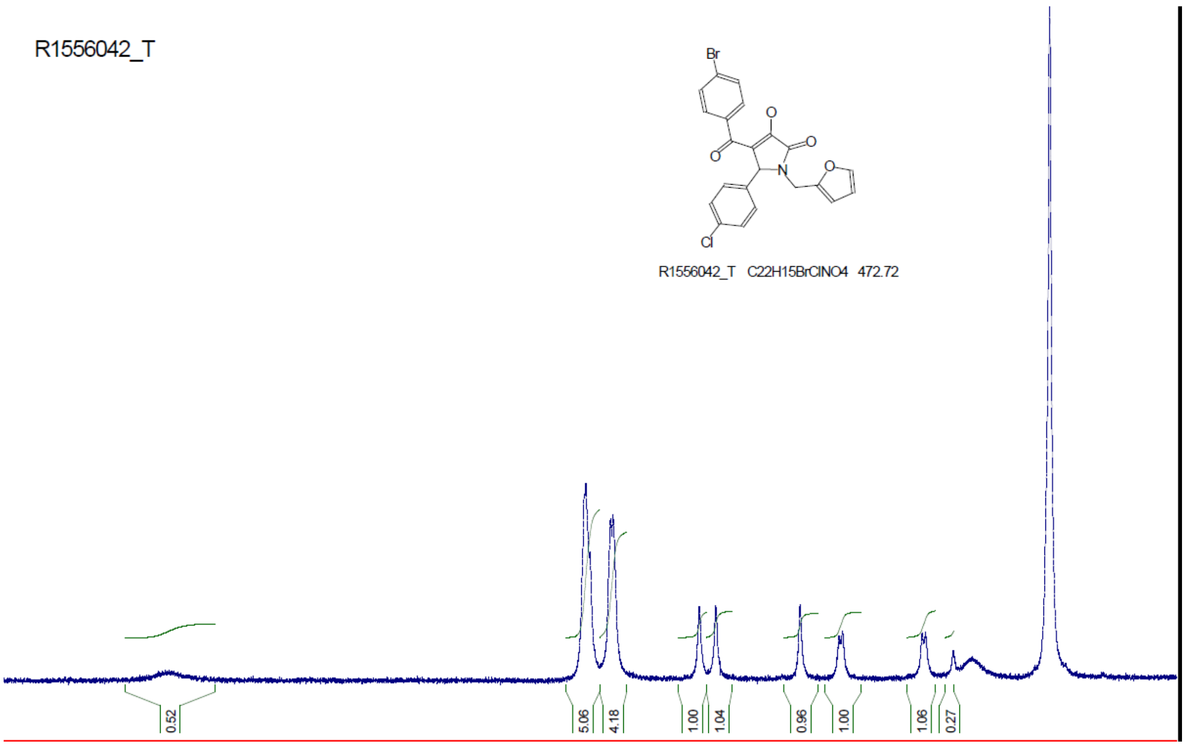
R1556042_T
Br
O
O
O
N
O
Cl
R1556042_T C22H15BrClNO4 472.72
0.52
5.06
4.18
1.00
1.04
0.96
1.00
1.06
0.27

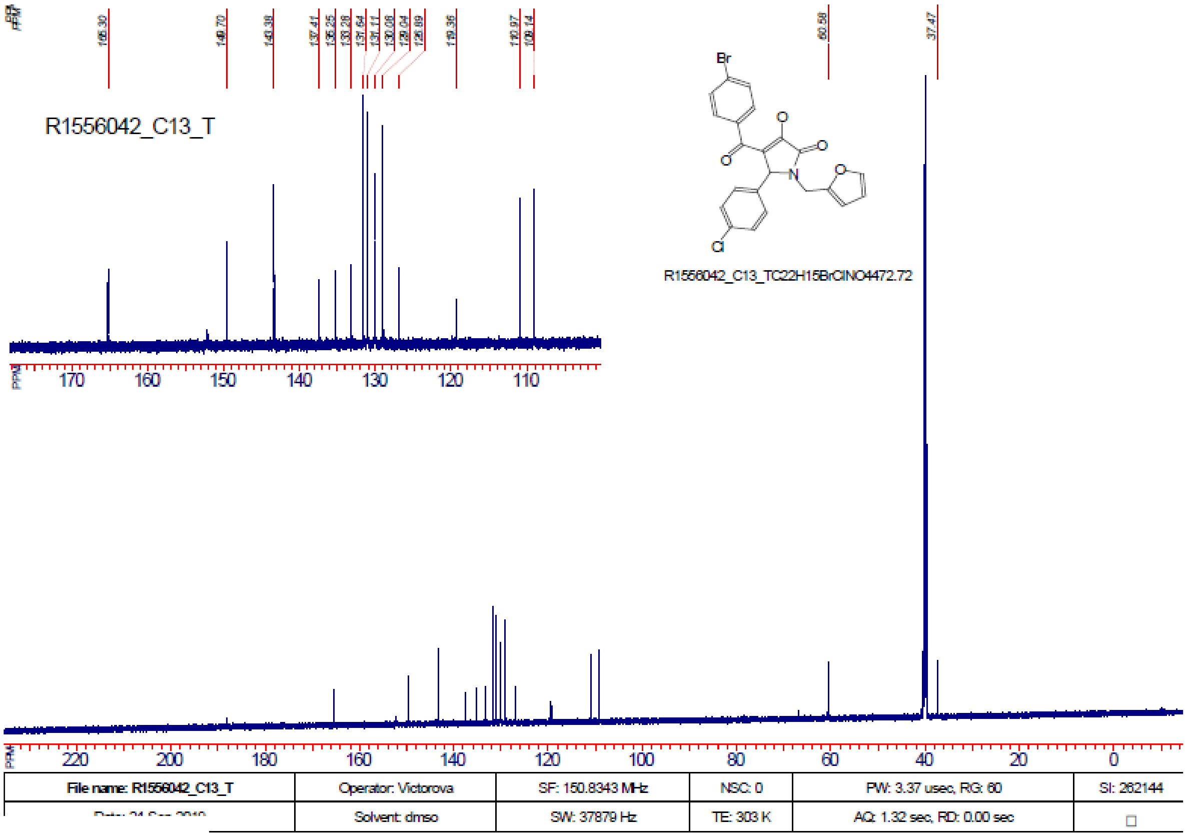
165.30
149.70
143.38
137.41
135.25
133.28
131.64
131.11
130.06
129.04
126.89
119.36
110.97
109.14
60.58
37.47
R1556042_C13_T
Br
O
O
O
N
O
Cl
R1556042_C13_TC22H15BrClNO4472.72
PPM
170 160 150 140 130 120 110
220 200 180 160 140 120 100 80 60 40 20 0
File name: R1556042_C13_T
Operator: Victorova
SF: 150.8343 MHz
NSC: 0
PW: 3.37 usec, RG: 60
SI: 262144
Solvent: dmso
SW: 37879 Hz
TE: 303 K
AQ: 1.32 sec, RD: 0.00 sec

Compound 42.

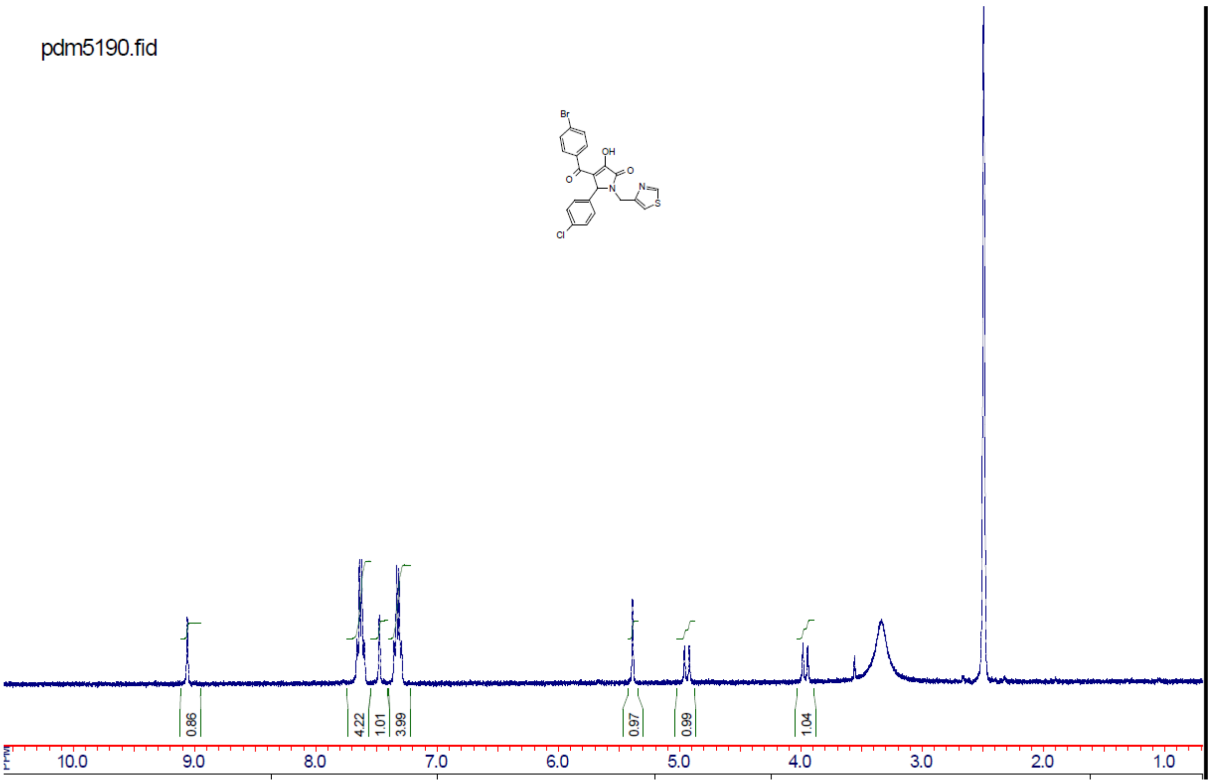
pdm5190.fid
Br
OH
O
N
S
Cl
0.86
4.22
1.01
3.99
0.97
0.99
1.04
PPM
10.0 9.0 8.0 7.0 6.0 5.0 4.0 3.0 2.0 1.0

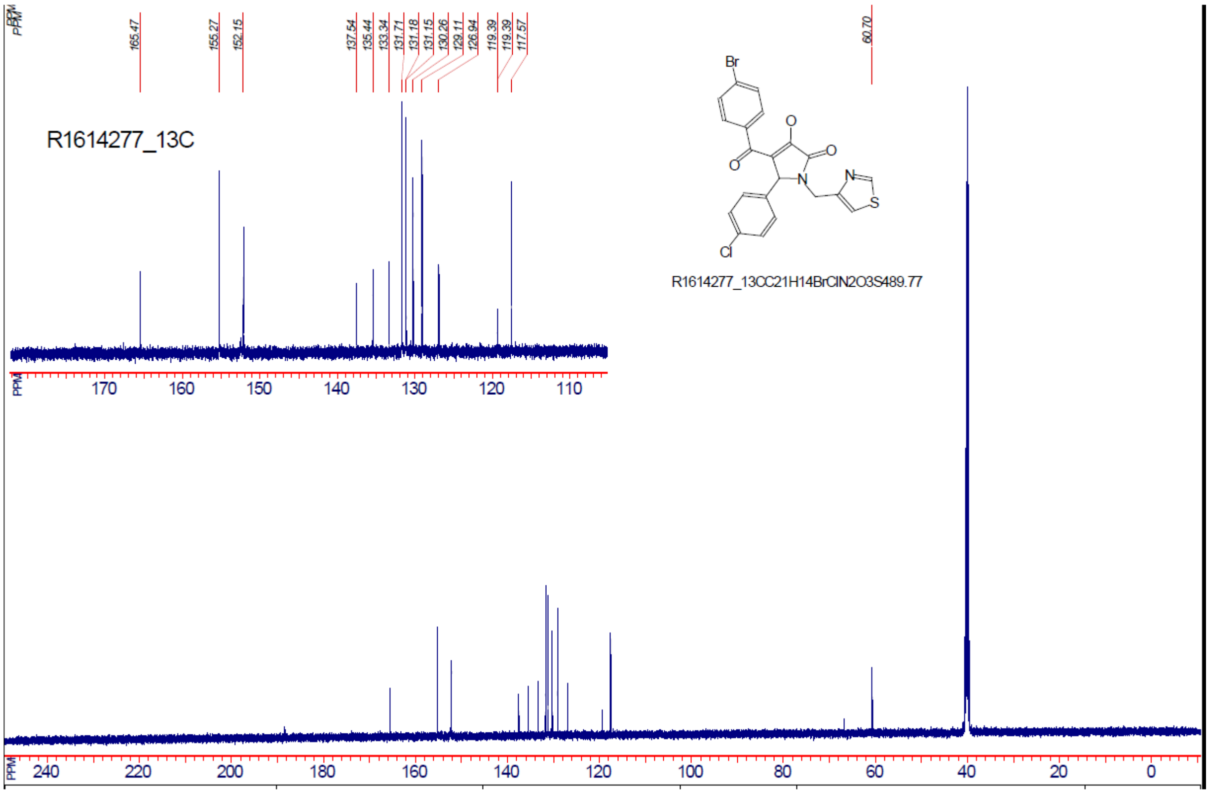
PPM
165.47 155.27 152.15 137.54 135.44 133.34 131.71 131.18 131.15 130.26 129.11 126.94 119.39 119.39 117.57 60.70
R1614277_13C
Br
O
O
O
N
N
S
Cl
R1614277_13CC21H14BrClN2O3S489.77
170 160 150 140 130 120 110
240 220 200 180 160 140 120 100 80 60 40 20 0

Compound 43.

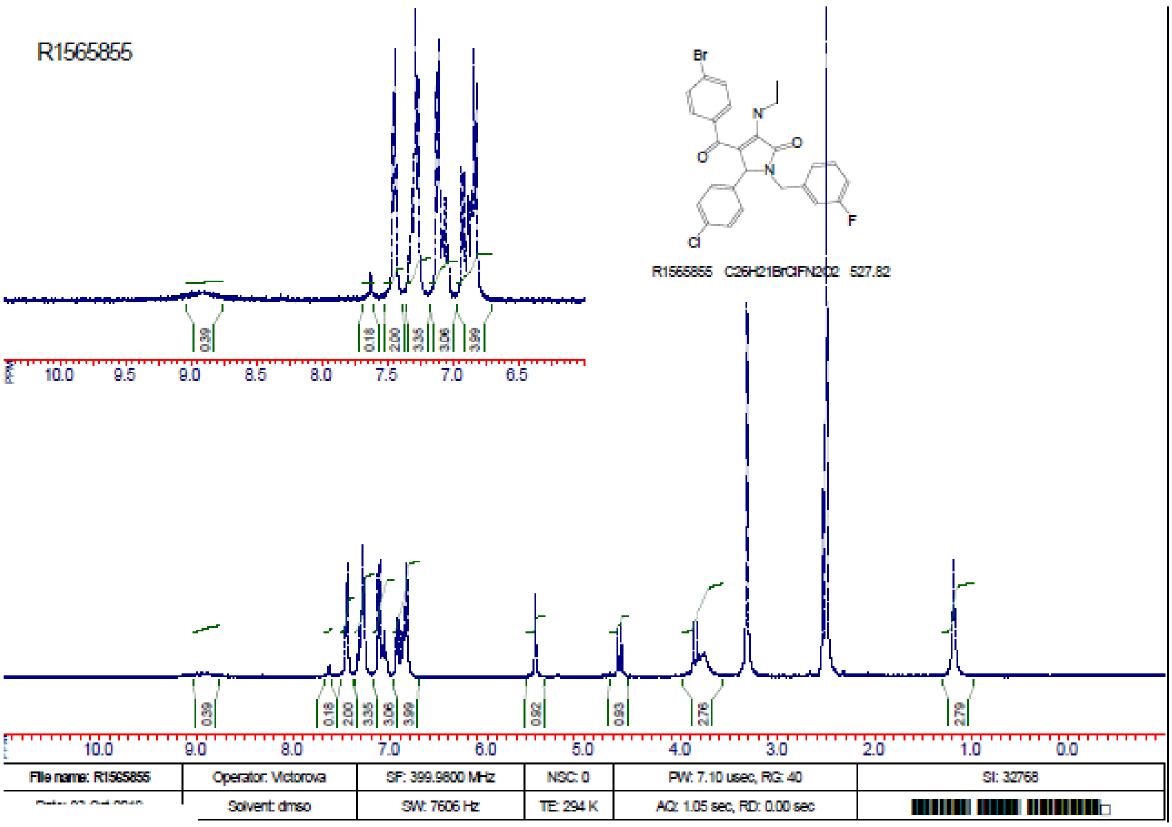

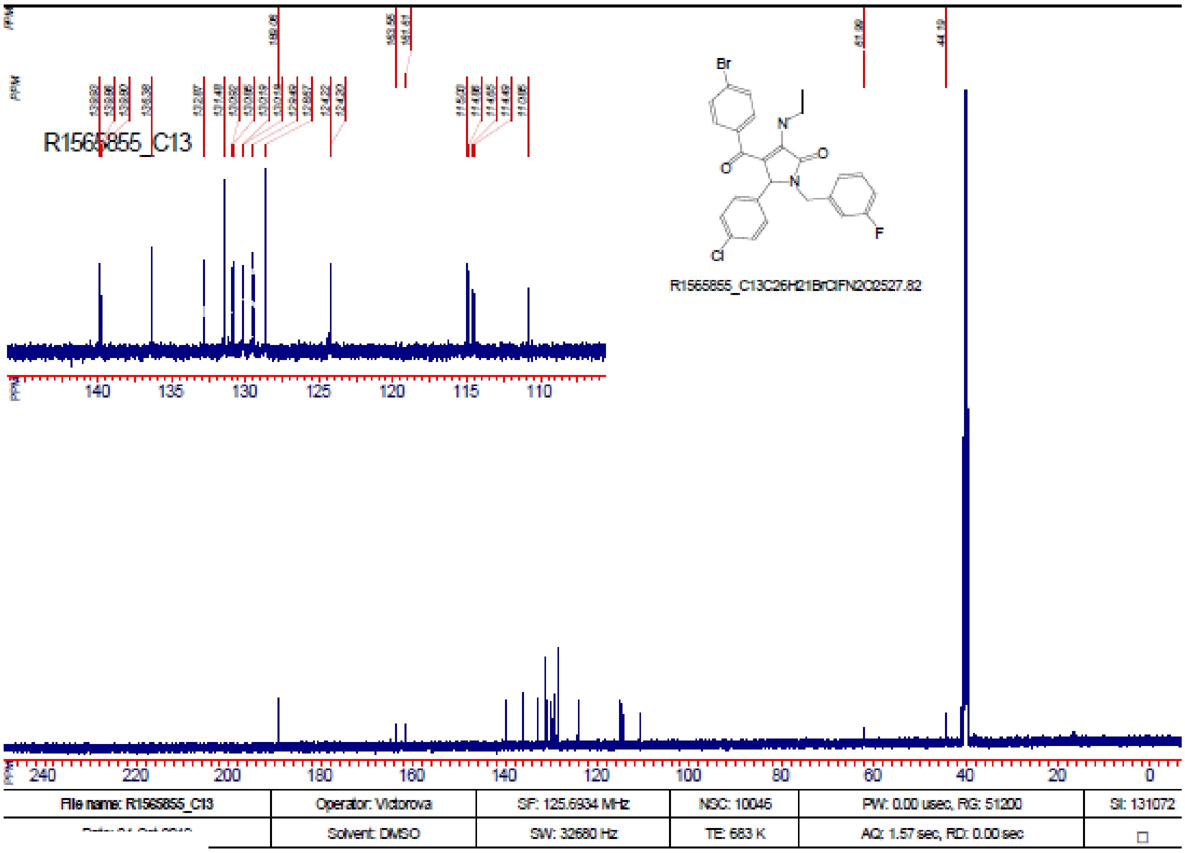

Compound 44.

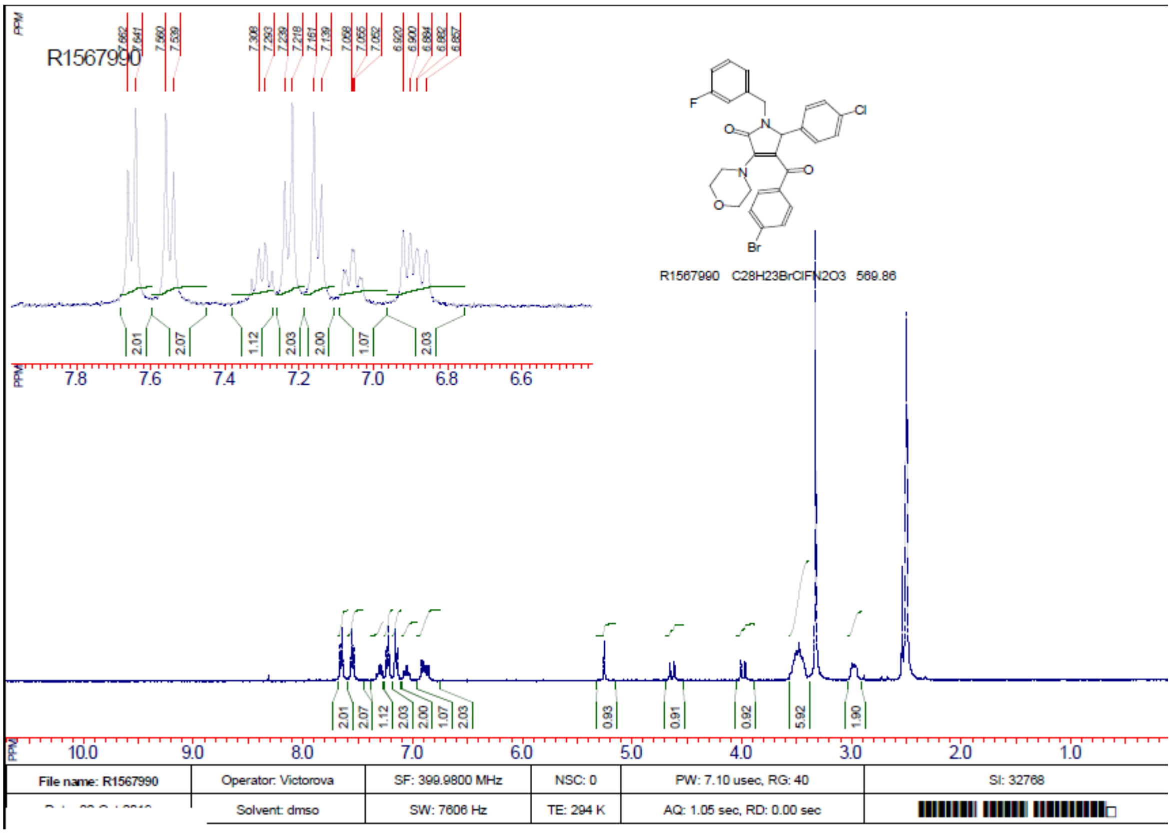

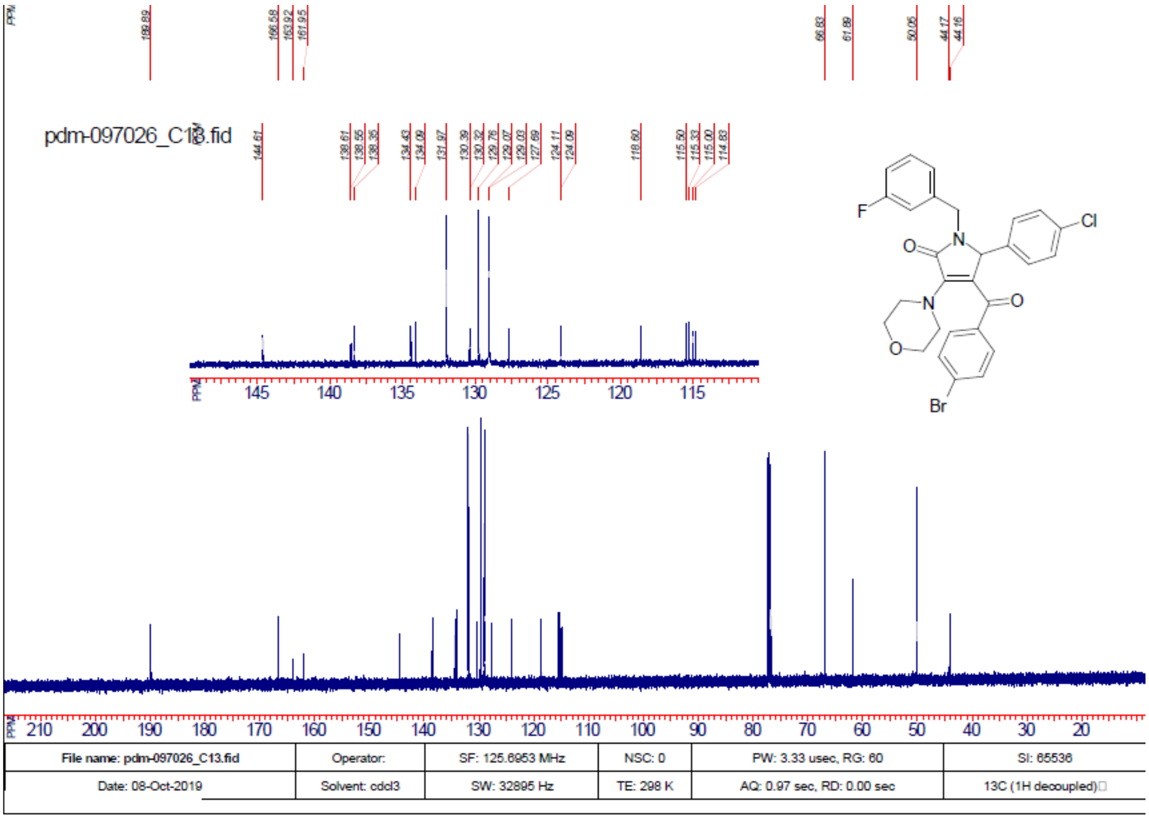

Compound 45.

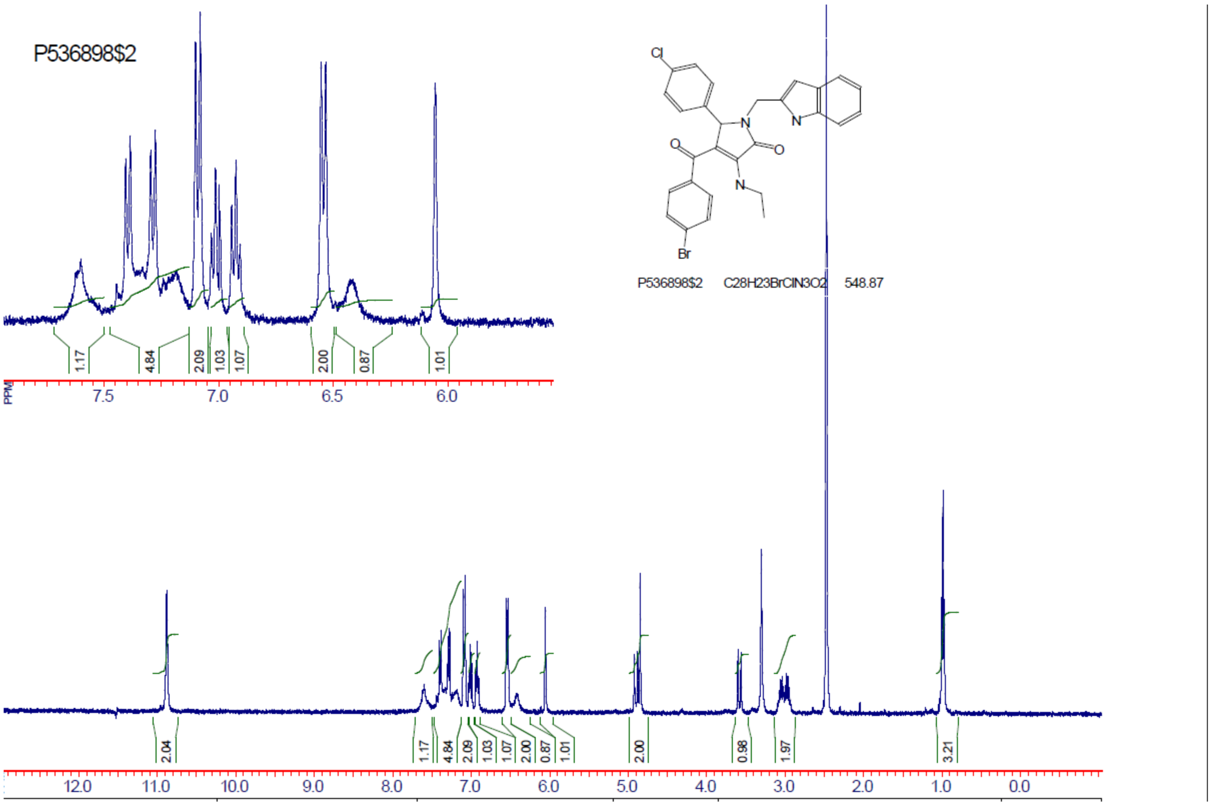
P536898$2
Cl
N
N
O
O
N
Br
P536898$2 C28H23BrClN3O2 548.87
1.17
4.84
2.09
1.03
1.07
2.00
0.87
1.01
PPM
7.5
7.0
6.5
6.0
2.04
1.17
4.84
2.09
1.03
1.07
2.00
0.87
1.01
2.00
0.98
1.97
3.21
12.0
11.0
10.0
9.0
8.0
7.0
6.0
5.0
4.0
3.0
2.0
1.0
0.0

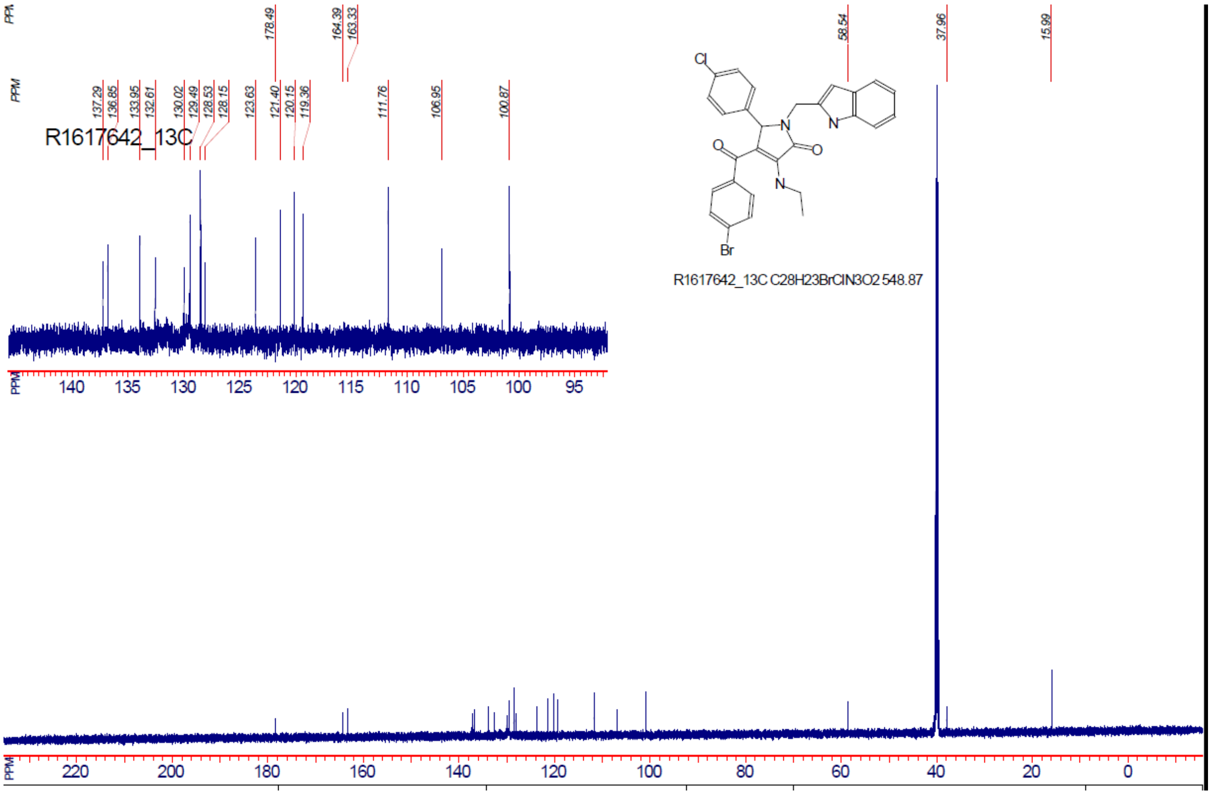
PPM
178.49
164.39
163.33
58.54
37.96
15.99
PPM
137.29
136.85
133.95
132.61
130.02
129.49
128.53
128.15
123.63
121.40
120.15
119.36
111.76
106.95
100.87
R1617642_13C
Cl
N
N
O
O
N
Br
R1617642_13C C28H23BrClN3O2 548.87
PPM
140
135
130
125
120
115
110
105
100
95
PPM
220
200
180
160
140
120
100
80
60
40
20
0
